# Association between higher serum uric acid levels and cognitive function: a systematic review and meta-analysis

**DOI:** 10.1093/gerona/glaf174

**Published:** 2025-08-09

**Authors:** Md Golam Rabbani, Sheikh M Alif, Zhen Zhou, Joanne Ryan, Md Nazmul Karim

**Affiliations:** School of Public Health and Preventive Medicine, Monash University, Melbourne, Victoria, Australia; School of Public Health and Preventive Medicine, Monash University, Melbourne, Victoria, Australia; Institute of Health and Wellbeing, Federation University Australia, Berwick, Victoria, Australia; School of Public Health and Preventive Medicine, Monash University, Melbourne, Victoria, Australia; School of Public Health and Preventive Medicine, Monash University, Melbourne, Victoria, Australia; School of Public Health and Preventive Medicine, Monash University, Melbourne, Victoria, Australia

**Keywords:** Hyperuricemia, Cognitive aging, Biomarkers, Metabolic syndrome, Oxidative stress

## Abstract

**Background:**

Serum uric acid (SUA) levels may be associated with cognitive function, but findings have been inconsistent, potentially varying by cognitive domain and sex. We aimed to determine the association of SUA and different domains of cognitive function.

**Methods:**

Five electronic databases were searched to identify relevant peer-reviewed articles. Studies investigating the association between SUA levels and cognitive function were included. Standardized mean difference (SMD) was calculated, and separate meta-analyses were conducted for each of the domains. Risk of bias was assessed using the Newcastle–Ottawa Quality Assessment Scale. Between-study heterogeneity was investigated through subgroup analysis and a meta-regression model using study-level covariates.

**Results:**

Ten prospective cohort and 16 cross-sectional studies were eligible for inclusion, but only a subset of these studies was included in each meta-analysis. Pooled estimates from cross-sectional studies showed that higher SUA levels were significantly associated with better global cognition (*n* = 6, SMD = 2.27, 95% CI, 1.18-3.35), and learning and memory (*n* = 4, SMD = 1.49, 95% CI, 1.12-1.87). Sensitivity analysis, excluding the study conducted on amyotrophic lateral sclerosis patients, resulted in better performance estimates for executive function (*n* = 4, SMD = 0.51, 95% CI, 0.47-0.55) and language (*n* = 2, SMD = 0.75, 95% CI, 0.71-0.79). The pooled result from 2 prospective cohort studies found a positive relationship between SUA levels and attention (SMD = 0.22, 95% CI, 0.07-0.36). Serum uric acid levels were associated with executive function and learning and memory in males, and with language in females.

**Conclusions:**

Higher SUA levels were associated with better global cognitive performance executive function, learning and memory, attention and language. These findings highlight low SUA levels as a potentially useful biomarker for cognitive decline.

## Introduction

Uric acid is a metabolic end product of purines. It plays a dual role in the body, acting as both a prooxidant and an antioxidant, highlighting the complex interplay of uric acid.^1–3^ Its oxidative stress properties contribute to the releases of reactive oxygen species,^4^ leading to chronic inflammation, and being associated with diseases, such as cancer, and cardiovascular diseases. On the other hand, the antioxidant capacity of uric acid mitigates oxidative stress, which may help slow the neurodegenerative process and delay the onset of cognitive dysfunction.^3^^,^^5^ As people age, cognitive function may change in various domains, including memory, motor function, language, attention, and executive function,^6^^,^^7^ and these changes may be early indicators of cognitive dysfunction.^8–10^

Associations between serum uric acid (SUA) and change in cognitive function have been reported in several observational studies,^11–19^ but the evidence is mixed, and may vary across cognitive domains. Understanding the link between SUA and cognitive function is important, given its potential for early intervention to slow decline before the progression to dementia.

A systematic review in 2022 highlighted the domain-specific impact of uric acid, reporting that higher SUA levels were associated with a faster decline in attention and executive function, while no significant associations were observed with global cognition, or learning and memory.^20^ However, this review had some methodological limitations and did not consider potential differences between males and females.

Males generally have higher SUA levels than females,^17^ and the impact of uric acid on various domains of cognitive function may differ by sex. Recent studies underscore the importance of examining sex-specific associations. For instance, a study reported that higher SUA levels were associated with better performance in the executive function in males but not in females.^16^ Similarly, another study observed that elevated SUA levels had beneficial effects on attention and processing speed in older males.^17^ A number of additional studies have been published since,^21–27^ highlighting the need for an updated review.

Therefore, we aimed to conduct a meta-analysis for each individual domain of cognitive function separately, to determine the domain-specific association by using available evidence. We also investigated the sex-stratified association for each of the domains of cognitive function for males and females separately.

## Methods

This review was conducted and reported following the ­Preferred Reporting Items for Systematic Reviews and Meta-­analyses guidelines.^28^ The protocol was registered with the International Prospective Register of Ongoing Systematic Reviews under registration numberCRD42024554258.

### Search strategy

Five electronic databases—MEDLINE (Ovid), EMBASE, CINAHL, Web of Science, and PubMed—were searched from inception to June 28, 2025 to identify relevant peer-reviewed publications. We also conducted a snowball search of the reference lists of relevant studies and used Google Scholar to identify additional studies. The search strategy included 2 key concepts: one for SUA and its closely related metabolites (urates), and one for cognitive function, neurocognitive dysfunction, neurocognitive domain, and cognitive impairment or dementia, combined with “OR” operators within concepts and “AND” operators to combine concepts as appropriate. For example, Medical Subject Headings terms for SUA included “uric acid,” “hyperuricemia,” and “gout,” and outcomes terms relating to both cognitive function/dysfunction, neurocognitive domains and cognitive impairment or dementia were included. The search strategy was developed for Ovid MEDLINE (using Medical Subject Headings terms) and then adapted for the other 4 databases (Table S4). All articles from the search were screened using Covidence.^29^

### Inclusion and exclusion criteria

Studies were eligible if they provided quantitative measures of the association between SUA and any domain of cognitive function among adults. Studies reported SUA levels as a categorical variable including binary groups (high and low), tertiles, quartiles, and quintiles were included. When assessing the outcomes, comparisons were made between the highest category within these groupings and the reference group. For continuous SUA measurements, associations with cognitive function were analyzed based on the effect of per mg/dL change in SUA levels. The outcomes of interest included domains of cognitive function such as global cognition, executive function, learning and memory, language, motor function, attention and social cognition, assessed using validated cognitive tests. Studies were excluded if they were animal studies, non-English articles, review articles, case reports, letters to the editor, or conference abstracts.

### Selection of studies

Two investigators (G.R. and S.A.) independently screened articles for eligibility. The first round involved screening the titles and abstracts followed by full-text screening. Disagreements were resolved through discussion with the third investigator (N.K.).

### Data extraction and study quality assessment

The data were extracted by G.R. using a data extraction table created in Microsoft Excel. Extracted data included study type, setting/country, sample size, participant characteristics (age, sex), length and frequency of follow-up, covariates, outcome variables, and key findings. A second reviewer (S.A.) randomly selected 25% of the included articles to extract and verify the accuracy of the data. The risk of bias for all included studies was evaluated using the Newcastle-Ottawa Quality Assessment Scale (NOS).^30^ Newcastle-Ottawa Quality Assessment Scale consists of 8 items categorized into 3 domains (selection, comparability, and outcomes) for cohort studies, and is adapted for cross-sectional studies (Table S1). High-quality studies are identified with stars. The higher the number of stars, the higher the quality of the study, with 9 stars being the maximum.^31^

### Statistical methods

Included studies used SUA levels as continuous, categorical, or both and reported results as β (beta) coefficients with 95% confidence interval (CI) or standard error (*SE*). For categorical SUA levels, the fully adjusted β coefficients were extracted with 95% CI or *SE* for the highest tertile, quartile, quintile, or high group (binary) and treating these as higher SUA levels and using the corresponding lower SUA levels as the reference group. For continuous SUA levels, we extracted the reported β coefficients with 95% CI or *SE*.

Cognitive function was assessed using different measurement scales, as shown in Table 1 (details in Table S2). We classified the cognitive function into 6 distinct cognitive domains: motor function, language, learning and memory, social cognition, attention, and executive function based on the Diagnostic and Statistical Manual of Mental Disorders fifth edition.^9^ In addition, we included an overall measure of global cognition. The majority of studies used multiple tests to assess a single domain, and different studies employed varying tests for the same domain. Raw mean differences can only be used when all studies in a meta-analysis apply the same measurement scale. If different studies use different instruments to assess the outcome, the measurement scales will vary, making it inappropriate to combine raw mean differences. In such cases, the mean difference in each study was divided by that study’s standard deviation to create an index—the standardized mean difference (SMD) that is comparable across studies.^43^^,^^44^ To address the inconsistencies, the SMD for the β coefficients was calculated. If the studies reported sex-stratified results, a fixed effects meta-analysis was conducted to estimate the overall effect size, which was then included in the meta-analysis as a single study.^43^^,^^45^ For studies performing multiple tests within one cognitive domain, pooling the results of different scores for a single domain could produce an inflated estimate. Therefore, an average SMD across these scores was included, ensuring that each study contributed only once to the final meta-analysis for a specific domain (Table S3). Considering the heterogeneity in reported results across studies, pooled estimates were calculated by combining the SMD using random-effect meta-analysis with restricted maximum likelihood methods. Separate meta-analyses were performed for each individual cognitive domain, stratified by the use of continuous and categorical SUA levels, and further divided into prospective cohort and cross-sectional studies.

**Table 1.** Summary characteristics of the included studies in the systematic review and meta-analysis.

Author (year)	Country	Sample size (male %)	Age (Mean ± *SD*)/Median (range)	Population	Cognitive function assessed	Measurement of cognitive function	Covariates adjusted	Key findings	Study quality (out of 9)
**Prospective cohort**									
**Jiang et al. 2024^21^**	China	7272 (49.59)	58.08 ± 8.53	General population	Global cognition	TICL, immediate and delayed word recall, figure drawing	Age, sex, BMI, education, marriage status, smoking status, alcohol consumption, physical activity and comorbidities	Elevated UA levels were associated with better global cognitive function	9
**Zhang et al. 2024^31^**	China	6236 (45.5)	58.3 ± 8.53	General population	Global cognition	(a) Time orientation, numerical ability, visual, and spatial abilities, including (TICS) and picture redrawing test and the season of the year and serial subtracting, (b) immediate and delayed word recall tasks.	Age, sex, marital status, educational level, smoking status, and drinking status, restriction on activities of daily living, BMI and other comorbidities	Higher SUA levels were associated with better global cognitive function and less subsequent cognitive decline among Chinese adults without hyperuricemia	9
**Huang et al. 2022^24^**	China	7828 (47.2)	58.1 ± 8.7	General population	(a) Global cognition, (b) executive function, (c) memory	Immediate and delayed word recall, time orientation, numerical ability and drawing of the overlapping pentagons	Age, sex, follow-up time, education, marital status, drinking status, smoking status, BMI, depressive symptoms, and other comorbidities	Low levels of SUA were associated with decline of global cognitive function	9
**Wang et al. 2022^32^**	China	3905 (50.4)	58.48 ± 8.54	Non-normotensive general population	(a) Global cognition, (b) episodic memory, (c) executive function	Immediate and delayed recall, figure drawing, time orientation, numerical ability	Age, gender, marital status, education level, smoking status, drinking status, depressive symptoms, BMI, and other comorbidities	Moderate increase of SUA was advantageous in improving global cognitive function or trajectories in a non-normotensive population	9
**Chen et al. 2021^26^**	China	3103 (45.6)	85.1 ± 11.7	General population	Global cognition	MMSE	Age, sex, education, drinking status, smoking status, marital status, regular exercise, BMI, and other comorbidities	High SUA levels were suggested the protective role in global cognitive decline among Chinese older adults	9
**Alam et al. 2020^33^**	United States	11 169 (41.3)	56.7 ± 5.7	General population	(a) Global cognition, (b) executive function, (c) memory, (d) language	DWRT, DSST, WFT	Age, sex, education, smoking status, waist-to-hip ratio, and other comorbidities	Higher SUA levels were associated with faster decline in global cognitive function, learning and memory and language function	9
**Kueider et al. 2017^34^**	United States	1451 (50)	Male: 65.8 ± 14. Female: 62.5 ± 13.7	General population	(a) Global cognition, (b) executive function, (c) memory, (d) attention, (e) language (f) visuospatial	(a) MMSE, (b) TMT-B, DS-backward task, (c) CVLT, (d) TMT-A, DS-forward, (e) Letter and category fluency, (f) clock 3:25, clock 11:10, and the card rotation tasks	Age, education, race, cardiovascular risk score	There were no significant associations between SUA and cognition in women. In men, higher SUA levels were associated with attenuated declines in attention and visuospatial abilities	8
**Beydoun et al. 2016^17^**	United States	2630 (43)	47 ± 0.3	General population	(a) Global cognition, (b) executive function, (c) memory, (d) attention, (e) language	(a) MMSE, (b) TMT-B, (c) CVLT, free delayed recall, (d) brief test of attention, (e) AF	Age, sex, race/ethnicity, marital status, education, poverty income ratio, current smoking status, BMI and other comorbidities	Higher SUA levels were associated with faster cognitive decline in a visual memory. However, increasing SUA levels were associated slower decline in attention/processing speed among older men	9
**Ye et al. 2016^35^**	South Korea	1064 (46)	73.7 ± 7.4	General population	Global cognition	MMSE	Age, education, race, BMI, and other factors and comorbidities	Higher SUA levels were associated with slower decline in global cognitive function, with a more pronounced effects in female	9
**Euser et al. 2009^15^**	The Netherlands	1724 (39)	64.1 ± 5.7	General population	(a) Global cognition, (b) executive function, (c) memory	(a) MMSE, (b) LDST, word fluency, Stroop test (reading, color naming and interference), (c) 15-WLT (immediate and delayed)	Age, sex, education, smoking status, and other comorbidities	Higher SUA levels were associated with better cognitive function	9
**Cross-sectional**									
**Tang et al. 2025^36^**	China	73 (63.0)	54.6 ± 10.1	Amyotrophic lateral sclerosis (ALS)	(a) Global cognition, (b) language, (c) verbal fluency, (d) executive function, (e) memory, (f) visuospatial functions	Edinburg cognitive and behavioral screen (ECAS)	Age, sex and education	Uric acid levels exhibited a positive correlation with executive function. However, no significant correlations were observed with global cognition, language ability, verbal fluency, memory, or visuospatial function	7
**Khaled et al. 2023^27^**	Qatar	931 (47.6)	48 (40-60)	General population	(a) Learning and memory (b) Speed-­response and movement	CANTAB-for (a) PAL and (b) RT	Age, sex, BMI, education and comorbidities	High SUA levels were associated with lower cognitive function in older adults	9
**Yuan et al. 2023^22^**	China	6509 (50)	57 (51-64)	General population	(a) Global cognition, (b) episodic memory, (c) mental intactness	Immediate and delayed words recall, TICL	Age, sex, BMI, education, smoking status and comorbidities	Lower SUA levels were associated with poorer cognitive performance compared to higher SUA levels	9
**Geng et al. 2022^23^**	United States	2763 (46.36)	≥60 (60-80)	General population	(a) Global cognition, (b) executive function, (c) memory, (d) speed/attention	CERAD word learning and recall modules, verbal fluency by the AF test, and the DSST	Age, sex, race, education, marital status, income, BMI, alcohol consumption, and other comorbidities	SUA levels were positively associated with cognitive function among older American adults	9
**Lee et al. 2021^25^**	South Korea	1343 (55)	73.9 ± 7.2	General population	(a) Global cognition, (b) executive function, (c) memory, (d) language, (e) visuospatial	MMSE, CDR, WMS	Age, education, BMI, baseline SUA, eGFR, and other comorbidities	Higher SUA levels were associated with significant cognitive decline in female	9
**Sun et al. 2020^37^**	China	274 (58)	Non-post stroke: 64.66 ± 11.57, Post Stoke: 71.30 ± 10.88	Acute cerebral infarction	Global cognition	MoCA	Sex, age, education, smoking status, alcohol consumption, and other comorbidities	Higher SUA levels were associated with post stoke cognitive decline	8
**Baena et al. 2017^16^**	Brazil	12 215 (45)	Male: (49.7 ± 7.5), Female: (50.2 ± 7.5)	General people (civil servant)	(a) Memory, (b) language, (c) executive function	(a) CERAD-WLMT-delayed recall, (b) SFT, (c) TMT	Age, education, smoking, BMI, alcohol consumption, depression, and other comorbidities	Higher SUA levels were associated with better performance on executive function in male but not in female	8
**Wang et al. 2017^11^**	China	12 798 (46)	59.3 ± 9.7	General population	(a) Global cognition, (b) episodic memory, (c) mental intactness	Immediate and delayed word recall, numeric ability, time orientation and picture drawing	Age, educational, depression scores, smoking, alcohol drinking, anxiety, obesity and other comorbidities	Higher SUA levels were associated with better cognition in later life but not associated with the rates of cognitive decline	9
**Liu et al. 2017^13^**	China	2102 (40.3)	71.2 ± 6.6	General population	Global cognition	MMSE	Age, education, marital status, BMI, current smoking, current drinking, physical activity, family history of dementia, hypertension, cerebral vascular disease and diabetes	Higher SUA levels were positively associated with cognitive function among elderly individuals in Chinese community; however, this association was not robust among participants with hyperuricemia	9
**Perna et al. 2016^38^**	Germany	1144 (43)	Male: 73.8 ± 2.7, Female: 74 ± 2.8	General population	Global cognition	COGTEL	Age, sex, educational level, BMI, smoking status, and alcohol consumption and other comorbidities	Higher SUA levels were negatively associated with cognitive function among female	9
**Molshatzki et al. 2015^14^**	Israel	446 (100)	62.3 ± 6.4	MI (male only)	(a) Global cognition, (b) executive function, (c) memory, (d) visuospatial	NCCB	Age, educational level, HDL cholesterol, chronic kidney disease, diabetes	Lower SUA levels in patients with pre-existing cardiovascular disease are associated with poorer cognitive function.	8
**Al-khateeb et al. 2015^39^**	Jordan	81 (62)	Not given	General population	Global cognition	MMSE	Not given	SUA levels were significantly lower in Alzheimer disease patients than control	7
**Vannorsdall et al. 2014^2^**	United States	436 (0)	73.9 ± 2.8 (70-79)	General population (female)	(a) Global cognition, (b) executive function, (c) memory, (d) attention, (e) language	(a) MMSE, (b) TMT-B, (c) HVLT-R learning, (d) DS, (e) letter fluency	Baseline SUA, demographic, and different health variables	Higher SUA levels were not associated with the decline of cognitive function	9
**Verhaaren et al. 2013^40^**	The Netherlands	814 (49.1)	62 ± 5.4	General population	(a) Global cognition, (b) executive function, (c) memory, (d) processing speed	(a) Neuropsychological test battery; (b and d) the Stroop test, LDST and word fluency test (animal categories), (c) 15-word verbal learning test (15-WLT)	Age, sex, BMI, alcohol consumption, smoking status, and other comorbidities	Higher SUA levels (hyperuricemia) were related to worse cognition	9
**Wu et al. 2013^41^**	China	2006	50-74	General population	Global cognition	MMSE	Age, sex, educational level, uric acid, BMI, smoking status, alcohol consumption, and other comorbidities	Higher SUA levels may play a protective role in cognitive decline	9
**Afsar et al. 2011^42^**	Turkey	247 (48)	60.5 ± 11	CKD	Global cognition	MMSE	Not given	SUA levels were independently and negatively associated with mild cognitive dysfunction in subjects with CKD.	6

Abbreviations: AF, Animal Fluency test; CANTAB, Cambridge neuropsychological test Automated Battery; CDR, Clinical Dementia Rating; CERAD, consortium to establish a registry for Alzheimer’s disease; CERAD-WLMT, CERAD-Word List Memory Test; CKD, chronic kidney disease; COGTEL, Cognitive Telephone Screening Instrument Test; CVLT, California verbal learning; DS, digit span; DSST, Digit Symbol Substitution Test; DWRT, Delayed Word Recall Test; HVLT-R, Hopkins Verbal Learning Test-Revised; LDST, letter digit substitution task; MI, myocardial infraction; MMSE, Mini-Mental State Examination; MoCA, Montreal Cognitive Assessment; NCCB, Neurotrax computerized cognitive battery; SFT, semantic fluency test; TICL, telephone interview cognitive status; TMT, Trail Marking Test; WAIS, Wechsler Adult Intelligence Scale; WFT, Word Fluency Test; WMS, Wechsler Memory Scale.

To standardize the units of SUA levels for the meta-analysis, we used mg/dL if reported in µmol/L are converted to mg/dL by multiplying 59.49. Sex-stratified estimates were generated to assess potential sex differentials in the association. A sensitivity analysis was conducted to test the robustness of the meta-analyses for general population, excluding populations with specific diseases [e.g., chronic kidney disease (CKD), acute cerebral infarction (ACI), myocardial infraction (MI), and amyotrophic lateral sclerosis (ALS)]. The percentage of variability across the studies attributable to heterogeneity beyond chance was estimated using the *I*^2^ statistic and Cochrane’s Q test. *I*^2^ values of 25%, 50%, and 75% were considered indicative of low, moderate, and high heterogeneity, respectively.^43^ A significance level *p* < .10 was used to determine the statistical significance of Cochrane’s Q.^43^ Subgroup analysis was performed to explore the sources of heterogeneity and was quantified using meta-regression models for study-level covariates: location (China vs outside China), sample size (≤1000 vs >1000), and age (≤60 years vs >60 years). Publication bias and small-study effects were examined using funnel plots and Egger’s test.^46^ Stata version 18.0 (Stata Corporation, College Station, TX) was used for statistical analyses.

## Results

**Figure 1.**
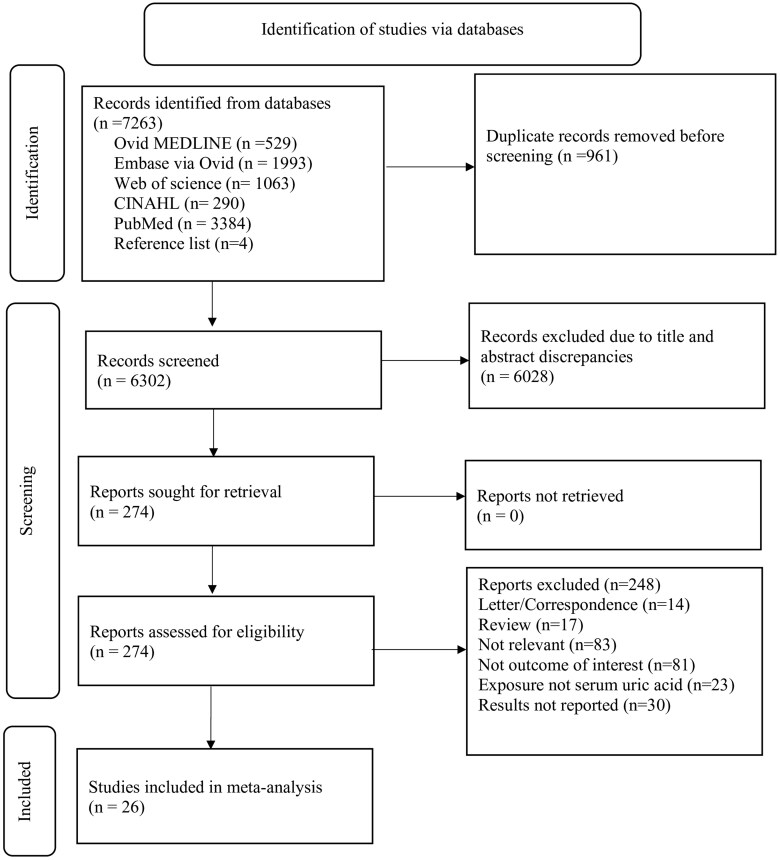
PRISMA (Preferred Reporting Items for Systematic Reviews and Mata-Analysis) 2020 flowchart diagram of the study selection process.

Our search identified 7263 articles, of which 274 were selected for full-text screening. A total of 26 studies met the eligibility criteria. The selection process is summarized in the Preferred Reporting Items for Systematic Reviews and Meta-analyses flow diagram (Figure 1).

### Study and population characteristics

The characteristics of the 26 included studies are summarized in Table 1. The sample sizes ranged from 73 to 12 798 participants, with mean ages spanning from 47 ± 0.3 to 85.1 ± 11.7 years (mean ± SD). A total of 24 studies reported global cognition, 13 reported executive function, 16 reported learning and memory, 7 each reported attention and language, 6 reported motor function, and 2 reported social cognition^11^^,^^22^ (detailed references in Table S2). Eleven studies were conducted in China, 5 in the United States, 2 in South Korea, and 1 each in 7 different countries. Of the included studies, 20 used data from prospective cohorts, although 5 of these reported cross-sectional analyses.^13^^,^^37^^,^^39^^,^^41^^,^^42^ Most studies sampled individuals from the general population, but 4 included individuals with a specific medical condition (ie, ACI, MI, ALS, and CKD).^13^^,^^36^^,^^37^^,^^42^ In the regression model, included studies commonly adjusted for age, sex, education, body mass index, and other relevant confounders, such as comorbidities, and lifestyle factors (eg, smoking and alcohol use, physical activity), as summarized in Table 1. All the studies included both male and female participants, except for 2 studies that focused on one sex.^2^^,^^14^

### Measurement of SUA and domains of cognitive function

All included studies used the standard spectrophotometry method (uricase enzymatic assay) to measure uric acid using fasting blood samples, except for 3 studies.^15^^,^^36^^,^^40^ Twelve studies modelled SUA levels as a continuous variable, 7 as a categorical variable, and 7 studies used it as both continuous and categorical variables to examine the association with cognitive domains.

Included studies measured 6 domains of cognitive function with different scales (Table S2). “Executive function” was measured by the Trail Marking Test-part B, Verbal Fluency, and Digit Span (DS)-backward. “Learning and memory” were evaluated by Immediate and Delayed word recall, the California Verbal Learning Test, and the Hopkins Verbal Learning Test—Revised. “Attention” was measured by the Trail Marking Test -part A, DS-forward, and Brief Test of Attention. “Language” was measured by Animal Fluency and Letter and Category Fluency. “Motor function” and “social cognition” were measured by Mini-Mental State Examination, Neurotrax Computerized Cognitive Battery, Picture complication/Block design, Time Orientation, and Numerical ability respectively. An overall measure—“global cognition” was measured either by Mini-Mental State Examination or by a composite score (average of z-score for each individual scale; standardized based on mean and *SD*). All the studies reported results in β coefficient with *SE* or 95% CI (Table S3).

### Study quality

Critical appraisal rating of the 26 included studies was conducted using the NOS scale, with ratings ranging from 6 to 9 stars with a median of 9 stars (Table 1 and Table S1). Based on the quality score of NOS, a total of 19 studies had a score of nine, 4 studies received 8 stars, 2 studies received 7 stars, and one study received 6 stars. Three studies received fewer than 8 stars, primarily because they adjusted only for age, sex, and education,^36^^,^^39^^,^^42^ without further adjustment for other potential confounders.

### Meta-analysis results

**Figure 2.**
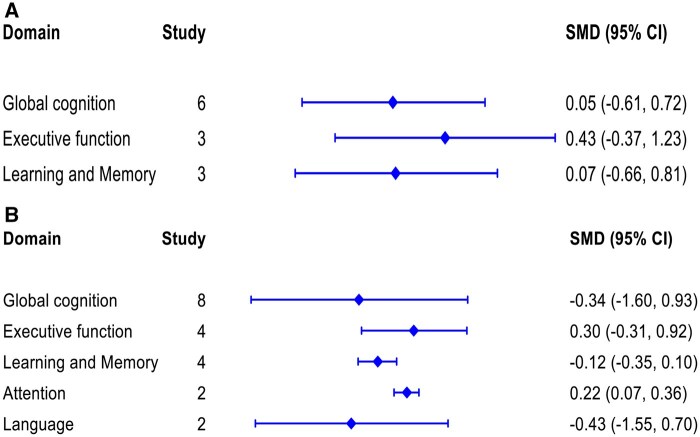
Summary results from all meta-analysis of prospective cohort studies for each of the individual cognitive domain. (A) SUA levels were used as a categorical variable (higher vs lower) and (B) SUA levels were used as a continuous variable. SUA = Serum uric acid.

**Figure 3.**
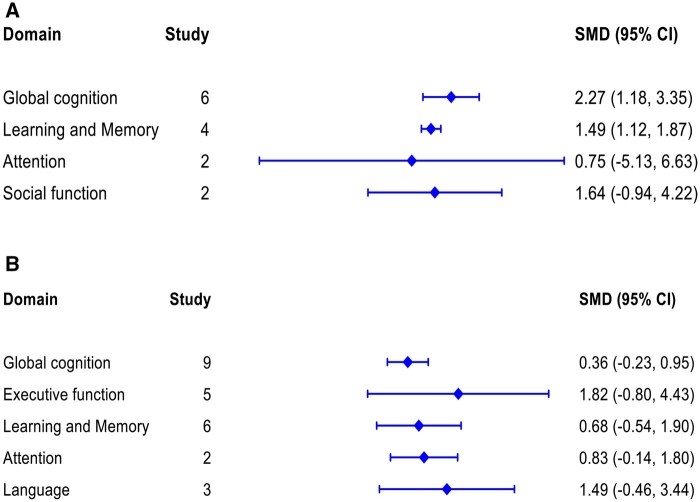
Summary results from all meta-analysis of cross-sectional studies for each of the individual cognitive domain. (A) SUA levels were used as a categorical variable (higher vs lower) and (B) SUA levels were used as a continuous variable. SUA = Serum uric acid

Separate meta-analyses were conducted for the different domains of cognitive function, stratified by study design (prospective cohort and cross-sectional studies). Figures 2 and 3 present summary results from all meta-analyses of prospective cohort and cross-sectional studies, using SUA levels as either a categorical and continuous variable.

#### Global cognition

**Figure 4.**
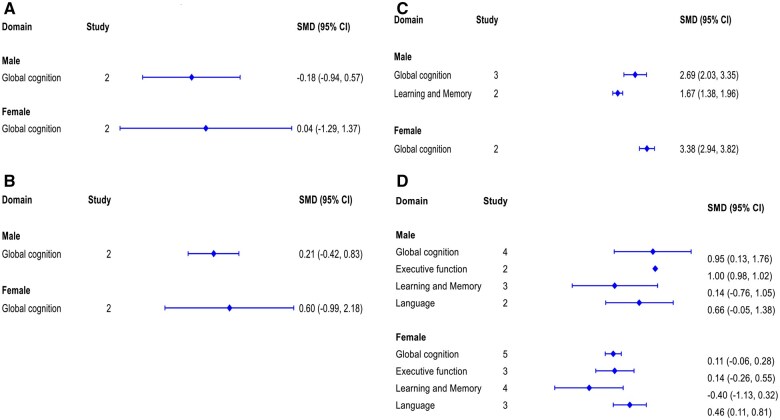
Sex-stratified summary of results from all meta-analysis for each individual cognitive domain. (A) Categorical SUA levels for prospective cohort studies, (B) Continuous SUA levels for prospective cohort studies, (C) Categorical SUA levels for cross-sectional studies, (D) Continuous SUA levels for cross-sectional; studies. SUA = Serum uric acid.

Summary meta-analysis results of 10 prospective cohort studies are presented in Figure 2A and B. The pooled effect size from 6 studies using categorical SUA levels showed no significant association between SUA and global cognition (SMD: 0.37, 95% CI, −0.33 to 1.07) (Figure 2A, Figure S1). A similar finding was observed for the pooled effect size from 8 studies using continuous SUA levels to examine global cognition (SMD: 0.74, 95% CI, –0.31 to 1.80) (Figure 2B, Figure S2). The sex-stratified analyses reported that the pooled estimates for both males and females were statistically insignificant (Figure 4A and B, Figures S1and S2). Based on subgroup analyses (test of group differences *p* < .001) and meta-regression (*p* < .001), study location (outside China) appears to be a significant source of heterogeneity. However, study sample size, participants’ age, and the measurement of global cognition were not associated with the pooled estimates (Figures S3 and S4). Egger’s tests indicated no statistically significant asymmetry in the funnel plot (*p* = .12 and *p* = .85), ruling out publication bias and small-study effects (Figures S3 and S4).

Summary meta-analysis results of 16 cross-sectional studies are presented in Figure 3A and B. The pooled effect size from 6 studies using categorical SUA levels showed that participants with higher SUA levels had significantly better global cognition (SMD: 2.27, 95% CI, 1.18-3.35) with a high heterogeneity across studies (Figure 3A and Figure S7) compared to those with lower SUA levels. However, the findings from 9 studies, those used continuous SUA levels, showed no significant association with global cognition (SMD: 0.36, 95% CI, −0.23 to 0.95) (Figure 3B and Figure S8). This result was influenced by the inclusion of studies conducted on ALS and CKD populations.^36^^,^^42^ After excluding these 2 studies from the meta-­analysis, the association became statistically significant (SMD: 0.62, 95% CI, 0.51-0.72), with no heterogeneity across the studies (Figure S9). The sex-stratified analyses showed that both males (SMD: 2.69, 95% CI, 2.03-3.35) and females (SMD: 3.38, 95% CI, 2.94-3.82) with higher SUA levels had better global cognition compared to those with lower SUA levels (Figure 4C and D and Figures S7 and S8). Sensitivity analyses, excluding 2 studies involving ACI^37^ and MI^14^populations, did not significantly alter the association (Figure S10). Based on subgroup analyses (test of group differences *p* < .001) and meta-regression (*p* < .001), study location, measurement of global cognition, and sample size appeared to be the sources of heterogeneity. However, participants’ age was not associated with the pooled estimates (Figures S11 and S12). Egger’s tests indicated no statistically significant asymmetry in the funnel plot (*p* = .79 and *p* = .41), ruling out publication bias and small-study effects (Figures S5 and S6).

#### Executive function

The effect sizes from both categorical and continuous SUA levels in prospective cohort and cross-sectional studies showed that SUA levels were not associated with improvement in executive function (Figures 2A and B and 3B, and Figures S15, S16, and S17A). However, after excluding a study conducted on ALS patients,^36^ the pooled estimate from 4 cross-sectional studies using continuous SUA levels showed a significant association (SMD: 0.51, 95% CI, 0.47-0.55), with no heterogeneity across the studies (Figure S17B). The sex-stratified analyses showed that an increase in SUA levels was significantly associated with better executive function performance in males (SMD: 1.00, 95% CI, 0.98-1.02) but not in females (Figure 4D and Figure S17A). Based on subgroup analyses and meta-­regression, study location and participants’ age appeared to be the sources of heterogeneity (Figures S18 and S19). Egger’s tests indicated no statistically significant asymmetry in the funnel plot (*p* = .96 and *p* = .67), ruling out publication bias and small-study effects (Figures S20 and S21).

#### Learning and memory

Summary meta-analysis results of 7 prospective cohort studies are presented in Figure 2A and B. The effect sizes from both categorical and continuous SUA levels were not associated with the improvement of learning and memory domain (Figure 2A and B, and Figures S22 and S23). However, the pooled estimate from 4 cross-sectional studies using categorical SUA levels showed a significant association with the learning and memory (SMD:1.49, 95% CI, 1.12-1.87), with no heterogeneity across the studies (Figure 3A and Figure S24). In contrast, the estimate from 6 cross-sectional studies using continuous SUA levels was not statistically significant (Figure 3B and Figure S25). The sex-stratified analyses reported that higher SUA levels were significantly associated with learning and memory performance in males (SMD: 1.67, 95% CI, 1.38-1.96) (Figure 4C and Figure S24). However, we did not perform a meta-analysis for females due to the inclusion of only one study. The sex-­stratified analyses using continuous SUA levels were not statistically ­significant (Figure 4D and Figure S25). Based on subgroup analyses and meta-regression, study sample size, study location, and participants’ age appeared to be unrelated to the pooled estimates (Figures S26–S28). Egger’s tests indicated no statistically significant asymmetry in the funnel plot (*p* = .78 and *p* = .75), ruling out publication bias and small-study effects (Figures S29 and S30).

#### Attention

A meta-analysis of 2 prospective cohort studies using SUA levels as a continuous variable showed better performance in the attention domain (SMD: 0.22, 95% CI, 0.07-0.36), with substantial heterogeneity across studies (Figure 2B and Figure S31). However, the pooled estimates from 2 cross-sectional studies, using categorical and continuous SUA levels separately, were not statistically significant (Figure 3A and B and Figures S32 and S33).

#### Language

Meta-analysis of both prospective cohort and cross-sectional studies showed that SUA levels were not significantly associated with performance in the language domain. (Figures 2B and 3B, Figures S34 and S35A). However, after excluding a study on ALS participants,^36^ the pooled estimate from 2 cross-sectional studies using continuous SUA levels showed a significantly altered association (SMD: 0.75, 95% CI, 0.71-0.79), with no heterogeneity across the studies (Figure S35B). The sex-­stratified analyses of 3 cross-sectional studies reported that an increase in SUA levels was significantly associated with performance in the language domain for females (SMD: 0.46, 95% CI, 0.11-0.81) but not for males (Figure 4D and S35B).

#### Motor function

We identified 5 studies that reported the association between SUA levels and the motor function domain.^2^^,^^14^^,^^25^^,^^34^^,^^36^ However, due to the variations in study design, the use of SUA levels either as categorical or continuous variable, and the inclusion of specific populations in these studies (eg, female only,^2^ or ALS patients only^36^) we did not perform a meta-analysis.

#### Social cognition

A meta-analysis of 2 cross-sectional studies showed that higher SUA levels were not associated with social cognition (Figure 3A and Figure S36), with a high heterogeneity across the studies. We did not conduct a meta-analysis for prospective cohort studies due to the availability of only one study.

## Discussion

We conducted separate meta-analyses for each cognitive domain, based on subsets of the 10 prospective cohort studies and 16 cross-sectional studies that reported outcomes related to cognitive function. Pooled estimates from cross-sectional studies indicated that higher SUA levels were associated with better performance in global cognition, executive function, learning and memory, and language. However, the estimates from prospective cohort studies showed a significant association only with attention. Sex-stratified analyses revealed that higher SUA levels were associated with better executive function, and learning and memory in males, whereas in females, the association was observed only with language.

In contrast to our findings, a meta-analysis by Tang et al.,^20^ which included 9 cross-sectional and 7 prospective cohort studies reported, no association between higher SUA levels and “global cognition” or “learning and memory” but found a relationship between higher SUA levels and decline in the combined “attention and executive function” domain. However, their analytical approach had key methodological limitations. Specifically, they used the lowest SUA quartile as the reference group and included multiple comparisons with each of the remaining quartiles. In contrast, our analysis included only one comparison per study, comparing the highest SUA group (eg, tertile/quartile/quintile) with the lowest, thereby maintaining statistical independence across studies and maximizing the exposure contrast. Additionally, although the previous review directly pooled β coefficients, we converted β coefficients into SMDs, enabling better comparability across studies using different cognitive assessment tools.

Another important distinction is that Tang et al.^20^ combined “executive function” and “attention” into a single domain. In our analysis, we treated these as separate cognitive domains in accordance with the Diagnostic and Statistical Manual of Mental Disorders fifth edition, which recognizes attention as a distinct cognitive domain. Analyzing these domains separately allows for a more precise and meaningful interpretation of the effects of SUA on specific cognitive functions. Collectively, these methodological differences likely contributed to the differing results between the 2 meta-analyses.

In our analysis using the continuous SUA levels, the association between SUA levels and global cognition was not statistically significant. However, after excluding studies involving populations with ALS and CKD, a significant positive association emerged. Since ALS and CKD are independent risk factors for cognitive dysfunction and dementia,^47–49^ their inclusion may have underestimated the pooled estimate. When examining categorical SUA levels, higher SUA levels were significantly associated with global cognition in the overall population, as well as in males and females separately. Notably, excluding a study on ACI from the overall analysis and another on MI from the male subgroup reduced the effect estimates but did not alter the direction or significance of the associations; underscoring the robustness of the categorical analysis. Since SUA is a known risk factor for ACI and MI, both of which are related to cognitive decline,^50–52^ one might expect these conditions to influence the findings. However, our results suggest that the significant association between higher SUA levels and global cognition persists even after accounting for these factors. This suggests that the relationship between SUA and cognitive function is not solely driven by the presence of ACI or MI but may reflect a more direct or independent effect of SUA on cognition. These findings highlight the importance of considering SUA as a potential biomarker for cognitive health, independent of its association with cardiovascular conditions.

Our meta-analysis of SUA levels and domains of cognitive function for prospective cohort studies and cross-sectional studies provided mixed results. The variation might be explained by the study design as a prospective cohort study reported the longitudinal change of the association, whereas cross-sectional analysis only provides a snapshot of the association at a single time point. Further, heterogeneity in the study population and outcome measurements may also affect the pooled estimates.

### Biological mechanisms

The mechanisms though which SUA influences different domains of cognitive function remain unclear. The dual role of uric acid underscores the importance of maintaining balanced levels to support cognitive function and overall brain health. Studies have shown that the progression of cognitive dysfunction is closely linked to oxidative stress, vascular inflammation, and inflammatory responses^53^^,^^54^ and that higher uric acid levels are associated with these mechanisms.^14^^,^^20^^,^^55^ However, uric acid also acts as a powerful antioxidant and may exert neuroprotective effects.^56^^,^^57^ The uric acid molecule generates superoxide (O2^−^) through enzymatic degradation of xanthine.^17^ Its antioxidant properties help in the removal of O2^−^ by preventing the degradation of superoxide dismutase enzyme.^58^ This removal of O2^−^ prevents its reaction with nitric oxide, which would otherwise lead to the formation of peroxynitrite (ONOO^−^), a biological oxidant associated with the pathology of many neurodegenerative diseases including multiple sclerosis,^59^ Parkinson’s disease,^60^ and Alzheimer’s disease.^61^

Our sex-stratified analysis yielded mixed results for both males and females. The variation in the effect of uric acid may be explained by physiological and hormonal functions. Lee et al.^25^ found that higher SUA levels may interact with apolipoprotein E4 to slow cognitive decline in females with mild cognitive impairment. In contrast, higher SUA levels were associated with white matter integrity only in males with Parkinson’s disease^62^ that cognitive vulnerability to oxidative stress may differ by sex. Additionally, cerebrovascular disease may confound the relationship between uric acid and cognitive decline in males, as males have a higher risk of cerebrovascular disease than females.^63^

Furthermore, higher plasma estrogen levels in females may lead to lower SUA levels through renal clearance.^64^^,^^65^ Research suggests that estrogen may influence the association between the urate transporter gene, SLC2A9, and SUA levels.^66^^,^^67^ Moreover, females appear to achieve better oxidative balance than males by enhancing mitochondrial respiratory chain function and antioxidant activities.^68^

### Bias and study quality of the systematic review and meta-analysis

Studies reported that high SUA levels are associated with obesity, alcohol consumption, smoking, physical activity, hypertension, diabetes, and other health conditions,^20^^,^^69^^,^^70^ all of which are also risk factors for cognitive dysfunction. Despite our selection of the most fully adjusted models, the potential for residual confounding could not be excluded, and inconsistent covariate adjustment across studies may have contributed to the observed heterogeneity in results. Nutritional status may also play a role in influencing both SUA levels and cognitive function. Research has shown that individuals with lower SUA levels tend to have poorer nutritional status,^71^^,^^72^ which increases the risk of cognitive impairment. Furthermore, since approximately one-third of total body urate originates from dietary sources,^73^ the influence of dietary patterns on SUA levels cannot be overlooked. A meta-analysis demonstrated that plant-based dietary patterns were associated with lower SUA levels, whereas animal-based dietary patterns were associated with higher SUA levels.^74^ Variation in dietary habits across countries, geographic regions, generations, and cultures—as well as changes in diet during long-term follow-up—could affect SUA levels. However, most of the included studies lacked data on dietary intake, which may have impacted the observed associations between SUA and cognitive function.

### Strengths and limitations of the review

We calculated SMD for each cognitive function and used ­random-effect models for the pooled estimates, which may have minimized the variability introduced by differences in study characteristics and methodologies. We conducted separate meta-analyses for prospective cohort and cross-sectional studies to quantify the pooled estimates, allowing us to compare the associations across 2 different study designs. Most of the included studies were based on general population-based longitudinal cohort or surveys from various countries, which enhanced to the generalizability of our findings. We also included studies with fully adjusted models to minimize confounding bias.

Although we included fully adjusted models in the meta-­analyses, the influence of residual confounders cannot be entirely excluded. A significant limitation of our findings is the substantial heterogeneity observed in each pooled estimate, which warrants caution in interpreting the results. Most studies reported baseline SUA levels without accounting for changes over time. Given that SUA concentrations may fluctuate over long follow-up periods, studies with repeated SUA measurements are warranted to better capture longitudinal trends. Some of the included studies, particularly cross-sectional studies, had a small sample size. As a result, the low statistical power may influence the ability to detect true effect sizes. Lastly, since most of our significant findings were derived from cross-sectional studies, the results do not provide a nuanced understanding of the temporal of casual nature of the association.

## Conclusion

Higher SUA levels were associated with better cognitive performance across several domains, including global cognition, executive function, learning and memory, attention, and language. Among males, significant associations were observed with executive function, and learning and memory, while in females, the association was noted only with language. Clinicians should consider that low SUA levels may serve as a potentially useful biomarker for cognitive decline. Randomized controlled trials are needed to confirm these associations and to understand the long-term and sex-specific effects of SUA on each domain of cognitive function.

## Supplementary Material

glaf174_Supplementary_Data

## Data Availability

Data will be made available upon request to the corresponding author.
