# Supplementary material for: Association between higher serum uric acid levels and cognitive function: a systematic review and meta-analysis
Source: J Gerontol A Biol Sci Med Sci. 2025 Aug 9;80(10):glaf174. doi: 10.1093/gerona/glaf174 (PMC12464678; doi:10.1093/gerona/glaf174)
Supplement: glaf174_Supplementary_Data [file glaf174_supplementary_data.pdf]

Supplementary Materials

Title: Association between higher serum uric acid levels and cognitive function: a systematic review and meta-analysis

Md. Golam Rabbani MPH [a], Sheikh M Alif PhD [a, b], Zhen Zhou PhD [a], Joanne Ryan PhD [a], Md. Nazmul Karim PhD [a]

Contents	Page
Table S1. Critical appraisal for 26 studies included in the meta-analysis by NOS.	3
Table S2. Cognitive domains and their respective functions of the 26 studies included in the meta-analysis.	4
Table S3: Extracted and calculated results (SMD) of the 26 studies included in the meta-analysis.	5-18
Table S4: Search Strategy of five elcetronic database.	19-21
Figure S1: Forest plot for the association between categorical SUA levels (higher vs lower) and global cognitive performance for prospective cohort studies.	22
Figure S2: Forest plot for the association between continuous SUA levels and global cognitive performance for prospective cohort studies.	23
Figure S3: Subgroup analysis for categorical SUA levels (higher vs lower) and global cognitive performance for prospective cohort studies.	24
Figure S4: Subgroup analysis for continuous SUA levels and global cognitive performance for prospective cohort studies.	25
Figure S5: Funnel plot for the association between categorical SUA levels (higher vs lower) and global cognitive performance for prospective cohort study.	26
Figure S6: Funnel plot for the association between continuous SUA levels and global cognitive performance for prospective cohort study.	26
Figure S7: Forest plot for the association between categorical SUA levels (higher vs lower) and global cognitive performance for cross-sectional studies.	27
Figure S8: Forest plot for the association between continuous SUA levels and global cognitive performance for cross-sectional studies.	28
Figure S9: Sensitivity analysis excluding two studies (from both sex; Tang et al. 2025 and Afsar et al.2011, population on ALS and CKD) for cross-section study of continuous SUA level and global cognitive performance.	29
Figure S10: Sensitivity analysis excluding one study from both sex (Sun et al.2020, population on Acute Cerebral Infarction) and one study from male (Molshatzki et al. 2015, population in Myocardial Infarction) for cross-section study for categorical SUA level (higher vs lower) and global cognitive performance.	30
Figure S11: Subgroup analysis for categorical SUA levels (higher vs lower) and global cognitive performance for cross-sectional studies.	31
Figure S12: Subgroup analysis for continuous SUA levels and global cognitive performance for cross-sectional studies.	32
Figure S13: Funnel plot for the association between categorical SUA levels (higher vs lower) and global cognitive performance for cross-sectional study.	33
Figure S14: Funnel plot for the association between continuous SUA levels and global cognitive performance for cross-sectional study.	33
Figure S15: Forest plot for the association between categorical SUA levels (higher vs lower) and executive function for prospective cohort studies.	34
Figure S16: Forest plot for the association between continuous SUA levels and executive function for prospective cohort studies.	34
Figure S17A: Forest plot for the association between continuous SUA levels and executive function for cross-sectional studies.	35
Figure S17B: Sensitivity analysis excluding a study (Tang et al. 2025; population on ALS) for cross-section study of continuous SUA level and executive function.	36
Figure S18: Subgroup analysis for continuous SUA levels and executive function for prospective cohort studies.	37

Figure S19: Subgroup analysis for continuous SUA levels and executive function for cross-sectional studies. 37
Figure S20: Funnel plot for the association between continuous SUA levels and executive function for prospective cohort study. 38
Figure S21: Funnel plot for the association between continuous SUA levels and executive function for cross-sectional study. 38
Figure S22: Forest plot for the association between categorical SUA levels (higher vs lower) and learning and memory for prospective cohort studies. 39
Figure S23: Forest plot for the association between continuous SUA levels and learning and memory for prospective cohort studies. 39
Figure S24: Forest plot for the association between categorical SUA levels (higher vs lower) and learning and memory for cross-sectional studies. 40
Figure S25: Forest plot for the association between continuous SUA levels and learning and memory for cross-sectional studies. 41
Figure S26: Subgroup analysis for continuous SUA levels and learning and memory for prospective cohort studies. 42
Figure S27: Subgroup analysis for categorical SUA levels (higher vs lower) and learning and memory for cross-sectional studies. 42
Figure S28: Subgroup analysis for continuous SUA levels and learning and memory for cross-sectional studies. 43
Figure S29: Funnel plot for the association between continuous SUA levels and learning and memory for prospective cohort study. 44
Figure S30: Funnel plot for the association between continuous SUA levels and learning and memory for cross-sectional study. 44
Figure S31: Forest plot for the association between continuous SUA levels and attention for prospective cohort studies. 45
Figure S32: Forest plot for the association between categorical SUA levels (higher vs lower) and attention for cross-sectional studies. 45
Figure S33: Forest plot for the association between continuous SUA levels and attention for cross-sectional studies. 46
Figure S34: Forest plot for the association between continuous SUA levels and language for prospective cohort studies. 47
Figure S35A: Forest plot for the association between continuous SUA levels and language for cross-sectional studies. 48
Figure S36B: Sensitivity analysis excluding a study (Tang et al. 2025; population on ALS) for cross-section study of continuous SUA level and language.5 49
Figure S36: Forest plot for the association between categorical SUA levels (higher vs lower) and social cognition domain for cross-sectional studies. 50
References 51-52

Table S1. Critical appraisal for 26 studies included in the meta-analysis by NOS.

Study reference	Selection	Comparability	Outcome / Exposure	Study quality (Out of 9)
Prospective Cohort				
Jiang (2024) (1)	✵✵✵✵	✵✵	✵✵✵	9
Zhang (2024) (2)	✵✵✵✵	✵✵	✵✵✵	9
Huang (2022) (3)	✵✵✵✵	✵✵	✵✵✵	9
Wang (2022) (4)	✵✵✵✵	✵✵	✵✵✵	9
Chen (2021) (5)	✵✵✵✵	✵✵	✵✵✵	9
Alam (2020) (6)	✵✵✵✵	✵✵	✵✵✵	9
Kueider (2017) (7)	✵✵✵✵	✵✵	✵✵	8
Beydoun (2016) (8)	✵✵✵✵	✵✵	✵✵✵	9
Ye (2016) (9)	✵✵✵✵	✵✵	✵✵✵	9
Euser (2009) (10)	✵✵✵✵	✵✵	✵✵✵	9
Cross-sectional				
Tang (2025) (11)	✵✵✵	✵	✵✵✵	7
Khaled (2023) (12)	✵✵✵✵	✵✵	✵✵✵	9
Yuan (2023) (13)	✵✵✵✵	✵✵	✵✵✵	9
Geng (2022) (14)	✵✵✵✵	✵✵	✵✵✵	9
Lee (2021) (15)	✵✵✵✵	✵✵	✵✵✵	9
Sun (2020) (16)	✵✵✵	✵✵	✵✵✵	8
Baena (2017) (17)	✵✵✵	✵✵	✵✵✵	8
Wang (2017) (18)	✵✵✵✵	✵✵	✵✵✵	9
Liu (2017)(19)	✵✵✵✵	✵✵	✵✵✵	9
Perna (2016) (20)	✵✵✵✵	✵✵	✵✵✵	9
Molshatzki (2015) (21)	✵✵✵	✵✵	✵✵✵	8
Al-khateeb (2015) (22)	✵✵✵✵	NA	✵✵✵	7
Vannorsdall (2014) (23)	✵✵✵✵	✵✵	✵✵✵	9
Verhaaren (2013) (24)	✵✵✵✵	✵✵	✵✵✵	9
Wu (2013) (25)	✵✵✵✵	✵✵	✵✵✵	9
Afsar (2011) (26)	✵✵✵	NA	✵✵✵	6

Table S2. Cognitive domains and their respective functions of the 26 studies included in the meta-analysis.

Cognitive domain	Tests used
Global cognition	MMSE (5, 7-10, 15, 19, 22, 23, 25, 26), MoCA (16) or by the average of the z-score of the individual test, the study was performed for cognitive function (1-4, 6, 13, 14, 18, 20, 21, 24) and ECAS (11).
Executive function	Verbal fluency (letter and category) (10, 14, 24), TMT-part B (7, 8, 17, 23) , DS: Backward (7, 8), DS-forward (8), Stroop Test (10, 24), Clock Drawing Command (8, 23), DSST (6), NCCB (21), Time orientation, numerical ability and drawing of the overlapping pentagons (3, 4) , MMSE (15) and ECAS (11).
Learning and Memory	CANTAB (12), Immediate and delayed words recall (3, 4, 6, 13, 18, 24), CERAD (word learning and recall modules, Immediate recall, delayed, retention/recognition test) (14, 17), CVLT (list-A, free delayed recall) (7, 8), HVLT-R (total learning, delayed recall) (23), NCCB (21), Benton visual retention test (8), MMSE, WMS (15) and ECAS (11).
Attention	CANTAB (12), TMT-part A (7, 8, 23), DS (forward) (7, 23), Brief test of attention (8, 23), DSST (14), Stroop (reading, color-naming) (24).
Language	AF (8, 23), Letter and Category fluency(7) , WFT (6), SFT (17), MMSE (15) and ECAS (11).
Motor function	MMSE (15), NCCB (21), Purdue pegboard (23), Clock 3:25, Clock 11:10 and the Card Rotation tasks (7) and ECAS (11).
Social cognition	TICL (13), Numeric ability, time orientation and picture drawing (18).

Notes: AF: Animal Fluency test, CANTAB: Cambridge Neuropsychological Test Automated Battery, CERAD: Consortium to Establish a Registry for Alzheimer's Disease, CERAD-WLMT =CERAD-Word List Memory Test, CKD: Chronic kidney disease , CDR: Clinical Dementia Rating, CVLT: California Verbal Learning, COGTEL: Cognitive Telephone Screening instrument Test, DWRT: Delayed Word Recall Test, DS: Digit Span, DSST: Digit Symbol substitution Test, HVLT-R: Hopkins Verbal Learning Test-Revised, LDST: Letter Digit Substitution Task , MMSE: Mini-Mental State Examination, MI: Myocardial Infraction, MoCA: Montreal Cognitive Assessment, NCCB: Neurotrax computerized cognitive Battery, SFT: Semanticfluen.cy test, TMT: Trail Marking test, TICL: Telephone interview cognitive status, WFT: Word Fluency Test, WAIS: Wechsler Adult Intelligence Scale, WMS: Wechsler Memory Scale, ECAS: Edinburg Cognitive and Behavioral Screen.

Table S3: Extracted and calculated results (SMD) of the 26 studies included in the meta-analysis.

Author (year)	Cognitive domain	Measurement of Cognitive function	Comparison (SUA)	Sex	Cognitive function	Risk estimate (β, 95%CI)	p-value	SE	SMD
**Prospective cohort study**									
Jiang (2024) (1)	Global Cognition	(a) by TICL, (b) by immediate and delayed word record, and c) by Figure drawing	Continuous	Both	Global cognition	0.09 [0.01, 0.17]	0.023	0.16	0.57
			Categorical (Q4 vs Q1)	Both	Global cognition	0.26 [0.02, 0.49]	0.022	0.46	0.56
Zhang (2024) (2)	Global Cognition	a) time orientation, numerical ability, visual, and spatial abilities, including (TICS) and picture redrawing test and the season of the year and serial subtracting, b) immediate and delayed word recall tasks.	Continuous	Both	Global cognition	0.026 [0.003, 0.049]	NA	0.05	0.58
Huang (2022) (3)	Global cognition	Immediate and delayed word recall, time orientation, numerical ability and drawing of the overlapping pentagons	Categorical [Low (2.76±0.54 mg/dl) vs Normal (5.1±0.76 mg/dl]	Both	Global cognition	0.24 [0.01, 0.47]	0.01	0.45	0.53
Wang (2022) (4)	Global Cognition	Summary of Episodic memory and Executive function	Continuous	Both	Global Cognition	0.07 (0.02, 0.13)	0.013	0.107	0.65

Table S3: Continued

Author (year)	Cognitive domain	Measurement of Cognitive function	Comparison (SUA)	Sex	Cognitive function	Risk estimate (β, 95%CI)	p-value	SE	SMD
**Prospective cohort study**									
Wang (2022) (4)	Global Cognition	Summary of Episodic memory and Executive function	Categorical [Q4 vs Q1 (mg/dl)]			0.33 (0.14, 0.52)	0.001	0.37	0.89
	Executive function	Immediate and delayed word recall,	Continuous		Executive function	0.077 (0.035, 0.12)	<0.001	0.083	0.92
			Categorical [Q4 vs Q1 (mg/dl)]			0.27(0.13, 0.41)	<0.001	0.27	0.98
	Learning and Memory	Time orientation, numerical ability and drawing of the overlapping pentagons	Continuous		Episodic memory	-0.005 (-0.033, 0.022)	0.71	0.05	-0.09
			Categorical [Q4 vs Q1 (mg/dl)]			0.063 (-0.030, 0.156)	0.18	0.19	0.32
Chen (2021) (5)	Global function	MMSE	Continuous	Both	Global function	0.64	0.001	0.17	3.69
			Categorical [Q4 vs Q1 (µmol/L)]			1.404	0.14	0.955	1.47

Table S3: Continued

Author (year)	Cognitive domain	Measurement of Cognitive function	Comparison (SUA)	Sex	Cognitive function	Risk estimate (β, 95%CI)	p-value	SE	SMD
**Prospective cohort study**									
Alam (2020) (6)	Global cognition	DWRT, DSST, WFT	Categorical [Q4 vs Q1 (µmol/L)]	Both	Global function	-0.15 [-0.25, -0.05]	0.02	0.20	-0.77
				Male		-0.15 [-0.29, -0.00]	NA	0.28	-0.53
				Female		-0.14[-0.26, -0.02]	NA	0.24	-0.60
	Executive function	DSST		Both	Executive function	-0.04[-0.11, 0.03]	0.28	0.14	-0.29
				Male		0.02[-0.13, 0.09]	NA	0.22	0.09
				Female		-0.04 [-0.12, 0.04]	NA	0.16	-0.25
	Learning and Memory	DWRT		Both	Memory	-0.23 [-0.38, -0.07]	0.007	0.30	-0.76
				Male		-0.25 [-0.47, -0.03]	NA	0.43	-0.58
				Female		-0.20 [-0.38, -0.02]	NA	0.35	-0.57
	Language	WFT		Both	Language	-0.08 [-0.15, -0.01]	0.004	0.14	-0.56
				Male		-0.05 [-0.17, 0.06]	NA	0.23	-0.24
				Female		-0.08 [-0.18, 0.01]	NA	0.18	-0.43

Table S3: Continued

Author (year)	Cognitive domain	Measurement of Cognitive function	Comparison (SUA)	Sex	Cognitive function	Risk estimate (β, 95%CI)	p-value	SE	SMD
**Prospective cohort study**									
Kueider (2017) (7)	Global cognition	MMSE	Continuous	Male	Global cognition	-0.002[-0.01, 0.01]	0.56	0.02	-0.10
				Female		-0.004 [-0.01, 0.01]	0.52	0.02	-0.20
	Executive Function	Trail Making Test<br>Part B and the Digit Span Backward task		Male	Executive Function	0.001 [-0.004, 0.007]	0.6	0.01	0.09
				Female		-0.002 [-0.009, 0.004]	0.44	0.01	-0.16
	Learning and Memory	California Verbal Learning<br>Test		Male	Memory	0.003 [-0.003, 0.01]	0.35	0.01	0.24
				Female		-0.002 [-0.01, 0.004]	0.46	0.01	-0.15
	Attention	Trail Making Test Part A<br>and the Digit Span Forward task		Male	Attention	0.006[0.0004, 0.01]	0.03	0.01	0.64
				Female		-0.002 [-0.006, 0.005]	0.94	0.01	-0.19
	language	letter and category<br>fluency		Male	Language	0.004 [-0.001, 0.009]	0.09	0.01	0.41
				Female		-0.001 [-0.006, 0.004]	0.8	0.01	-0.10
	Motor function	Clock 3:25, Clock 11:10 and the Card Rotation tasks.		Male	Visuospatial	0.007 [0.001, 0.01]	0.01	0.01	0.79
				Female		-0.001 [-0.007, 0.01]	0.83	0.02	-0.06

Table S3: Continued

Author (year)	Cognitive domain	Measurement of Cognitive function	Comparison (SUA)	Sex	Cognitive function	Risk estimate (β, 95%CI)	p-value	SE	SMD
**Prospective cohort study**									
Beydoun (2016) (8)	Global cognition	MMSE, b), c) CVLT, free delayed recall, d), e) AF	Continuous	Both	Global cognition	-0.01	0.45	0.01	-1
	Executive function	DS-forward			Executive function	-0.01	0.19	0..01	-1
	Executive function	DS-backward			Executive function	-0.01	0.63	0.02	-0.5
	Executive function	Clock command			Executive function	0.01	0.86	0.02	0.05
	Executive function	TMT-B			Executive function	0.51	0.35	0.55	0.93
	Learning and Memory	CVLT- list A			Memory	-0.01	0.64	0.03	-0.33
	Learning and Memory	CVLT-free delayed recall			Memory	-0.01	0.55	0.01	-1
	Learning and Memory	Benton visual retention test			Memory	0.07	0.001	0.02	3.5
	Attention	Brief test of attention			Attention	-0.01	0.27	0.01	-1
	Attention	TMT-A			Attention	0.23	0.22	0.19	1.21
	Language	AF			Language	-0.01	0.78	0.01	-1

Table S3: Continued

Author (year)	Cognitive domain	Measurement of Cognitive function	Comparison (SUA)	Sex	Cognitive function	Risk estimate (β, 95%CI)	p-value	SE	SMD
**Prospective cohort study**									
Ye (2016) (9)	Global cognition	MMSE	Continuous	Male	Global cognition	0.06 [0.005, 0.12]	0.03	0.11	0.53
				Female		0.18 [0.11, 0.24]	<0.001	0.13	1.41
		MMSE	Categorical [T3 vs T2 (mg/dl)]	Male		0.09 [-0.10, 0.28]	0.34	0.37	0.24
				Female		0.33 [0.11, 0.55]	0.003	0.43	0.77
Euser (2009) (10)	Global cognition	a) MMSE, b) LDST, Word fluency, Stroop test (reading, color naming and interference), c) 15-WLT (immediate and delayed)	Continuous	Both	Global cognition	2.38 [0.0, 4.16]	NA	4.08	0.58
	Executive function	LDST, Word fluency, Stroop test (reading, color naming and interference),			Executive function	1.19 [-1.19, 3.57]	NA	4.66	0.26
	Learning and Memory	15-WLT (immediate and delayed)			Memory	3.57 [1.19, 6.54]	NA	5.25	0.68

Table S3: Continued

Author (year)	Cognitive domain	Measurement of Cognitive function	Comparison (SUA)	Sex	Cognitive function	Risk estimate (β, 95%CI)	p-value	SE	SMD
**Cross-sectional Study**									
Tang (2025) (11)	Global cognition	ECAS	Continuous	Both	Global cognition	0.14 [-0.24, 0.93]	0.248	0.11	1.22
	Executive function	ECAS			Executive function	0.232 [0.004, 0.053]	0.023	0.05	4.83
	Memory	ECAS			Memory	0.091[-0.014, 0.017]	0.452	0.03	2.99
	Language	ECAS			language	0.100 [-0.012, 0.018]	0.407	0.03	3.40
	Motor function	ECAS			Visuospatia l function	0.151 [-0.001, 0.006]	0.211	0.007	22.01
Khaled (2023) (12)	Learning and Memory	CANTAB	Categorical [High (≥360μmol/L) vs Normal (<369 μmol/L)]	Both	Memory	-6.3 [-11.77, -1.45]	<0.001	10.11	-0.63
	Attention	CANTAB			Attention	-74 [-208.81, 61.81]	<0.001	265.21	-0.28
Yuan (2023) (13)	Global cognition	Immediate and delayed words recall, TICL	Categorical [Q4 vs Q1 (mg/dl)]	Both	Global cognition	-0.42 [-0.73, -0.11]	0.004	0.61	-0.69
	Learning and Memory	Immediate and delayed words recall			Episodic Memory	-0.31 [-0.53, -0.09]	0.003	0.43	-0.72
	Social Cognition	TICL			Mental Intactness	-0.11 [-0.28, 0.06]	0.159	0.33	-0.33

Table S3: Continued

Author (year)	Cognitive domain	Measurement of Cognitive function	Comparison (SUA)	Sex	Cognitive function	Risk estimate (β, 95%CI)	p-value	SE	SMD
**Cross-sectional Study**									
Geng (2022) (14)	Global cognition	CERAD word learning and recall modules, verbal fluency by the AF test, and the DSST	Continuous	Both	Global Cognition	0.08[0.02, 0.14]	0.01	0.11	0.75
			Categorical [Q4 vs Q1(μmol/L)]			0.29 [0.06, 0.52]	0.05	0.45	0.64
	Executive function	Verbal fluency by the AF test	Continuous	Both	Executive Function	0.17[0.01,0.33]	0.05	0.31	0.54
			Categorical [Q4 vs Q1(μmol/L)]			0.67 [0.04, 1.27]	0.05	1.21	0.56
	Learning and Memory	CERAD word learning and recall modules	Continuous	Both	Memory	0.17 [-0.01, 0.35]	0.1	0.36	0.49
			Categorical [Q4 vs Q1(μmol/L)]			0.28 [-0.43, 0.99]	0.46	1.39	0.20
	Attention	DSST	Continuous	Both	Processing speed	0.42[0.30, 0.80]	0.05	0.50	0.84
			Categorical [Q4 vs Q1(μmol/L)]			2.25 [0.72, 3.78]	0.01	3.0	0.75
	Executive function	Time orientation, numerical ability and drawing of the overlapping pentagons			Executive function	0.24 [0.07, 0.41]	<0.001	0.33	0.72
	Memory	Immediate and delayed word recall			Memory	0.03 [0.08, 0.14]	0.69	0.06	0.51

Table S3: Continued

Author (year)	Cognitive domain	Measurement of Cognitive function	Comparison (SUA)	Sex	Cognitive function	Risk estimate (β, 95%CI)	p-value	SE	SMD
**Cross-sectional Study**									
Lee (2021) (15)	Global cognition	MMSE, CDR, WMS	Continuous	Male	Global Cognition	0.05	0.96	0.03	1.67
				Female		-0.001	0.08	0.02	-0.04
	Executive function	MMSE		Male	Executive function	0.01	0.01	0.01	1
				Female		0.002	0.15	0.01	0.33
	Learning and Memory	MMSE, WMS		Male	Memory	0.01	0.01	0.01	1
				Female		0.002	0.24	0.01	0.4
	Language	MMSE		Male	Language	0.01	0.01	0.01	1
				Female		0.004	0.14	0.01	0.67
	Motor function	MMSE	Continuous	Male	Visuospatia l	0.003	0.31	0.01	0.5
				female		-0.01	0.66	0.01	-1.0
			Categorical [Q4 vs Q1 (1µmol/L)]			1.4	0.14	0.96	1.47
Sun (2020) (16)	Global cognition	MoCA	Categorical [High (>6mg/dl) vs low (<6mg/dl)]	Both	Global cognition	0.007	NA	0.002	3.5

Table S3: Continued

Author (year)	Cognitive domain	Measurement of Cognitive function	Comparison (SUA)	Sex	Cognitive function	Risk estimate (β, 95%CI)	p-value	SE	SMD
**Cross-sectional Study**									
Baena (2017) (17)	Executive function	TMT-B	Continuous	Male	Executive function	-3.11 [-4.56, -1.62]	NA	2.91	-1.07
				Female		-0.09 [-1.71, 1.53]	NA	3.18	-0.03
	Learning and Memory	CERAD-WLMT- (Immediate recall)	Continuous	Male	Memory	-0.1[-0.18, -0.02]	NA	0.15	-0.63
				Female		-0.06 [-0.15, 0.02]	NA	0.16	-0.41
		CERAD-WLMT- (Delayed recall)		Male		-0.4[-0.08, -0.00]	NA	0.08	-0.51
				Female		-0.21 [-0.06, 0.02]	NA	0.08	-2.58
		Retention/recognition test		Male		-0.02[-0.04, -0.00]	NA	0.04	-0.61
				Female		-0.02 [-0.37, -0.00]	NA	0.36	-0.06
	Language	SFT	Continuous	Male	Language	0.06[-0.05, 0.16]	NA	0.20	0.27
				Female		0.03 [-0.09, 0.14]	NA	0.22	0.12

Table S3: Continued

Author (year)	Cognitive domain	Measurement of Cognitive function	Comparison (SUA)	Sex	Cognitive function	Risk estimate (β, 95%CI)	p-value	SE	SMD
**Cross-sectional Study**									
Wang (2017) (18)	Global cognition	Immediate and delayed word recall, Numeric ability, time orientation and picture drawing	Continuous	Male	Global cognition	0.02	0.58	0.03	0.67
				Female		0.01	0.75	0.04	0.25
			Categorical [Q4 vs Q1]	Male		0.58	0.01	0.23	2.52
				Female		0.77	0.001	0.23	3.35
	Learning and Memory	Immediate and delayed word recall,	Continuous	Male	Episodic memory	-0.00	0.99	0.03	0
				Female		0.00	0.99	0.03	0
			Categorical [Q4 vs Q1]	Male		0.25	0.09	0.15	1.67
				Female		0.29	0.05	0.15	1.93
	Social cognition	Numeric ability, time orientation and picture drawing	Continuous	Male	Mental Intactness	0.01	0.6	0.02	0.5
				Female		0.01	0.44	0.02	0.5
			Categorical [Q4 vs Q1]	Male		0.35	0.14	0.14	2.5
				Female		0.48	<0.001	0.14	3.43

Table S3: Continued

Author (year)	Cognitive domain	Measurement of Cognitive function	Comparison (SUA)	Sex	Cognitive function	Risk estimate (β, 95%CI)	p-value	SE	SMD
**Cross-sectional Study**									
Liu (2017) (19)	Global cognition	MMSE	Continuous	Male	Global cognition (Hyperuricemia)	-0.03 [-0.56, 0.39]	0.72	0.93	-0.03
				Female		-0.01[-0.43, 0.37]	0.88	0.78	-0.01
			Categorical [Q4 vs Q1]	Male		0.99 [0.87, 1.13]	0.54	0.25	3.9
				Female		1.0[0.92, 1.18]	0.92	0.25	3.92
		MMSE	Continuous	Male	Global cognition (non-hyperuricemia)	0.14 [0.04, 0.42]	0.05	0.37	0.38
				Female		0.28 [0.02, 0.53]	0.04	0.50	0.56
			Categorical [Q4 vs Q1]	Male		1.65 [1.12, 2.43]	0.03	1.28	1.29
				Female		1.92 [1.02, 3.35]	0.02	2.28	0.84
Perna (2016) (20)	Global cognition	COGTEL	Continuous	Both	Global cognition	-0.32 [-0.68, 0.05]	NA	0.72	-0.45
				Male		-0.12 [-0.64, 0.39]	NA	1.01	-0.12
				Female		-0.57 [-1.10, -0.04]	NA	1.04	-0.55
Molshatzki (2015) (21)	Global cognition	NCCB	Categorical [Higher (combined of top four quintiles) vs bottom (Q1 mg/dl)]	Male	Global cognition	3.77	0.001	1.29	2.92
	Executive function	NCCB			Executive function	2.62	0.02	1.43	1.83
	Memory	NCCB			Memory	4.38	0.01	1.82	2.40
	Motor function	NCCB			Visuospatial	3.31	0.08	2	1.66

Table S3: Continued

Author (year)	Cognitive domain	Measurement of Cognitive function	Comparison (SUA)	Sex	Cognitive function	Risk estimate (β, 95%CI)	p-value	SE	SMD
**Cross-sectional Study**									
Al-khateeb (2015) (22)	Global cognition	MMSE	Continuous	Both	Global cognition	-0.042 [-1.54, 1.17]	NA	2.66	-0.02
Vannorsdall (2014) (23)	Global cognition	MMSE	Continuous	Female	Global cognition	0.01	0.84	0.07	0.14
	Executive function	TMT-part B			Executive function	-0.1	0.68	0.24	0.42
	Executive function	Clock drawing, command			Executive function	-0.04	0.56	0.08	0.5
	Executive function	Clock drawing, copy			Executive function	0.07	0.19	0.06	-1.17
	Learning and Memory	HVLT-R learning			Memory	-0.13	0.53	0.21	-0.62
	Learning and Memory	HVLT-R recall			Memory	-0.05	0.64	0.11	-0.45
	Attention	DS			Attention	-0.03	0.87	0.16	0.19
	Attention	Brief test of attention			Attention	-0.18	<0.05	0.08	2.25
	Attention	TMT-part A			Attention	-0.11	0.84	0.54	0.20
	Attention	Pattern comparison test			Attention	-0.43	0.14	0.29	1.48
	Language	Letter fluency			Language	-0.03	0.93	0.39	0.08
	language	AF			Language	-0.15	0.44	0.19	0.79
	Motor function	Purdue pegboard			Purdue pegboard	0.64	<0.07	0.35	-1.83

Table S3: Continued

Author (year)	Cognitive domain	Measurement of Cognitive function	Comparison (SUA)	Sex	Cognitive function	Risk estimate (β, 95%CI)	p-value	SE	SMD
**Cross-sectional Study**									
Verhaaren (2013) (24)	Global cognition	a) Neuropsychological test battery; c) 15-word verbal learning test(15-WLT), b & d) the Stroop test, LDST and word fluency test (animal categories)	Continuous	Both	Global cognition	-4.76 [-8.33, -1.78]	NA	6.41	-0.74
	Executive function	the Stroop test, LDST and word fluency test (animal categories)			Executive function	-5.35 [-8.92, -1.78]	NA	6.70	-0.77
	Learning and Memory	immediate and delayed recall of the 15-WLT			Memory	-4.16 [-8.92, 0.59]	NA	9.33	-0.45
	Attention	Stroop reading, Stroop color-naming, The LDST			Processing Speed	-5.35[-9.52, -1.78]	NA	7.58	-0.71
Wu (2013) (25)	Global cognition	MMSE	Categorical [T3 vs T1 (µmol/L)]	Both	Global cognition	0.002	0.022	0.001	2
Afsar (2011) (26)	Global cognition	MMSE	Continuous	Both	Global cognition	-0.30[-0.42, -0.12]	<0.001	0.29	-1.02

Notes: AF: Animal Fluency test, CANTAB: Cambridge Neuropsychological Test Automated Battery, CERAD: Consortium to Establish a Registry for Alzheimer's Disease, CERAD-WLMT =CERAD-Word List Memory Test, CKD: Chronic kidney disease , CDR: Clinical Dementia Rating, CVLT: California Verbal Learning, COGTEL: Cognitive Telephone Screening instrument Test, DWRT: Delayed Word Recall Test, DS: Digit Span, DSST: Digit Symbol substitution Test, HVLT-R: Hopkins Verbal Learning Test-Revised, LDST: Letter Digit Substitution Task , MMSE: Mini-Mental State Examination, MI: Myocardial Infraction, MoCA: Montreal Cognitive Assessment, NCCB: Neurotrax computerized cognitive Battery, SFT: Semanticfluen.cy test, TMT: Trail Marking test, TICL: Telephone interview cognitive status, WFT: Word Fluency Test, WAIS: Wechsler Adult Intelligence Scale, WMS: Wechsler Memory Scale, ECAS: Edinburg Cognitive and Behavioral Screen.

Table S4: Search Strategy of five elcetronic database.

Database	Concept A	Concept B	Remarks
	Serum Uric Acid	Cognitive function, neurocognitive domain, cognitive dysfunction, cognitive impairment or dementia	
MEDLINE	Subject headings: Uric Acid/ or Hyperuricemia/ or Gout/	Subject Headings: Dementia/ or Dementia, Vascular/ or Frontotemporal Dementia/ or Dementia, Multi-Infarct/ or Parkinson Disease/ or Alzheimer Disease/ or Nervous System Diseases/ or Ischemic Stroke/ or Lewy Body Disease/ or Kluver-Bucy Syndrome/ or Diffuse Neurofibrillary Tangles with Calcification/ or Cognition Disorders/ or Cognitive Dysfunction/ or Cognition/ or Neurocognitive Disorders/ or Memory Disorders/ or Nerve Degeneration/ or Frontotemporal Lobar Degeneration/	Total: 529
	Keywords: ((serum level* or plasma level* or blood level*) adj2 uric acid).mp. OR ((serum level* or plasma level* or blood level*) adj2 urate).mp. OR (hyperuric?emia or gout).mp	Keywords: (((neurocogniti* or cogniti* or memor*) adj (disorder* or dysfunction* or disease* or decline* or impair*)) or (dementia* or alzheimer* or huntington* disease*) or cognitive function).mp	
EMBASE	Subject Headings: uric acid/ or uric acid blood level/ or hyperuricemia/ or gout	Subject Headings: dementia/ or multiinfarct dementia/ or frontotemporal dementia/ or mixed dementia/ or frontal variant frontotemporal dementia/ or Alzheimer disease/ or Parkinson disease/ or diffuse Lewy body disease/ or diffuse neurofibrillary tangles with calcification/ or Huntington chorea/ or Kluver Bucy syndrome/ or progressive nonfluent aphasia/ or "disorders of higher cerebral function"/ or cognition/ or cognitive defect/ or mild cognitive impairment/	Total: 1993
	Key words: ((serum level* or plasma level* or blood level*) adj2 uric acid).mp. OR ((serum level* or plasma level* or	Keywords: (((neurocogniti* or cogniti* or memor*) adj (disorder* or dysfunction* or disease* or decline* or impair*)) or (dementia* or alzheimer* or huntington* disease*) or cognitive function).mp.	

	blood level*) adj2 urate).mp. OR (hyperuric?emia or gout).mp		
CINAHL	(MH "Uric Acid"), (MH "Hyperuricemia") , (MH "Gout")	(MH "Dementia") OR (MH "Frontotemporal Dementia+") OR (MH "Dementia, Vascular+") OR (MH "Dementia Patients") OR (MH "Dementia, Multi-Infarct") OR (MH "Delirium, Dementia, Amnestic, Cognitive Disorders+") OR (MH "Lewy Body Disease") OR (MH "Kohlschutter-Tonz Syndrome") OR (MH "Dementia, Presenile+") OR (MH "Dementia, Senile+")<br>(MH "Cognition") OR (MH "Cognition Disorders+")<br>(MH "Mild Cognitive Impairment")<br>(MH "Alzheimer's Disease") OR (MH "Lewy Body Disease") OR (MH "Neurodegenerative Diseases+"), (MH "Parkinson Disease")<br>(MH "Mixed Dementias"), (MH "Huntington's Disease")<br>(MH "Memory Disorders"), (MH "Nerve Degeneration"), (MH "Frontotemporal Lobar Degeneration")	Total: 290
	((“serum level*” or “plasma level*” or “blood level*”) N2 “uric acid”) OR ((“serum level*” or “plasma level*” or “blood level*”) N2 “urate”) OR (hyperuric?emia or gout)	((neurocogniti* or cogniti* or memor*) N0 (disorder* or dysfunction* or disease* or decline* or impair*))<br>or (dementia* or alzheimer* or “huntington* disease*” or “cognitive function”)	
Web of Science	“Uric Acid” or Hyperuricemia or Gout	Dementia or “Dementia, Vascular” or “Frontotemporal Dementia” or “Dementia, Multi-Infarct” or “Parkinson Disease*” or “Alzheimer Disease*” or “Nervous System Diseases* or “Ischemic Stroke” or “Lewy Body Disease” or “Kluver-Bucy Syndrome” or “Diffuse Neurofibrillary Tangles with Calcification” or “Cognition Disorders” or “Cognitive Dysfunction” or Cognition” or “Neurocognitive Disorders” or “Memory Disorders” or “Nerve Degeneration” or “Frontotemporal Lobar Degeneration”	Total: 1063
	(("serum level*" or "plasma level*" or "blood level*")	((((neurocogniti* or cogniti* or memor*) “NEAR” (disorder* or dysfunction* or disease* or decline* or impair*)) or (dementia* or alzheimer* or huntington* disease*) or "cognitive function"))	

	“NEAR/2” "uric acid")<br>(("serum level*" or "plasma level*" or "blood level*")<br>“NEAR/2” urate)		
PubMed	((((((((((((((((((((((((("uric acid") OR (hyperuricemia)) OR ("serum uric acid")) OR ("uric acid blood level")) OR ("plasma uric acid")) OR ("plasma urate")) OR (gout)) AND (dementia)) OR ("Dementia, Vascular")) OR ("Frontotemporal Dementia")) OR ("Dementia, Multi-Infarct")) OR ("Parkinson Disease")) OR ("Alzheimer Disease")) OR ("Nervous System Diseases")) OR ("Lewy Body Disease")) OR ("Kluver-Bucy Syndrome")) OR ("Diffuse Neurofibrillary Tangles with Calcification")) OR ("Cognition Disorders")) OR ("Cognitive Dysfunction")) OR ("Cognition")) OR ("Neurocognitive Disorders")) OR ("Memory Disorders")) OR ("Nerve Degeneration")) OR ("Frontotemporal Lobar Degeneration")) OR ("mild cognitive impairment")) OR ("cognitive defect")= 3384		

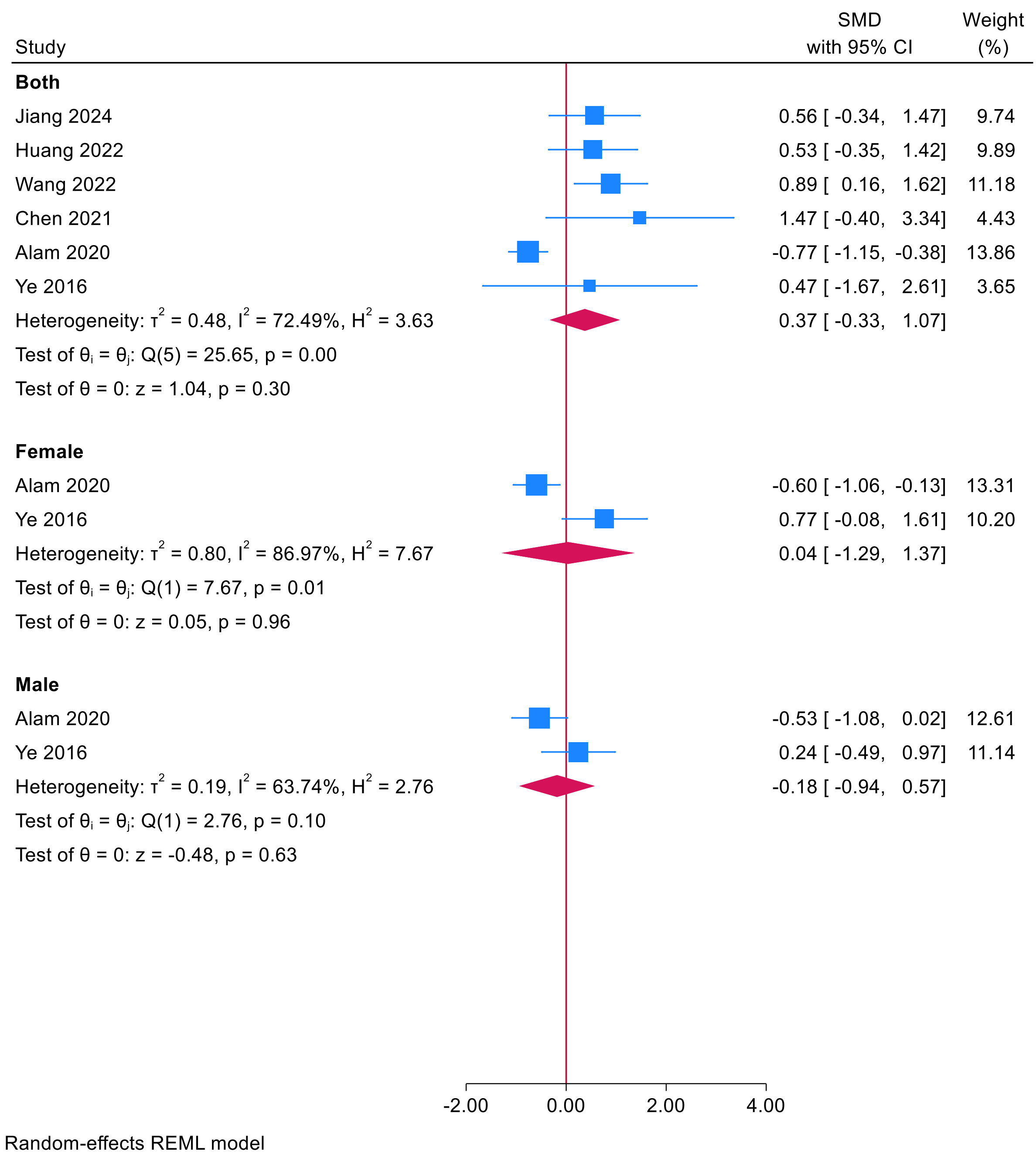

Figure S1: Forest plot for the association between categorical SUA levels (higher vs lower) and global cognitive performance for prospective cohort studies.

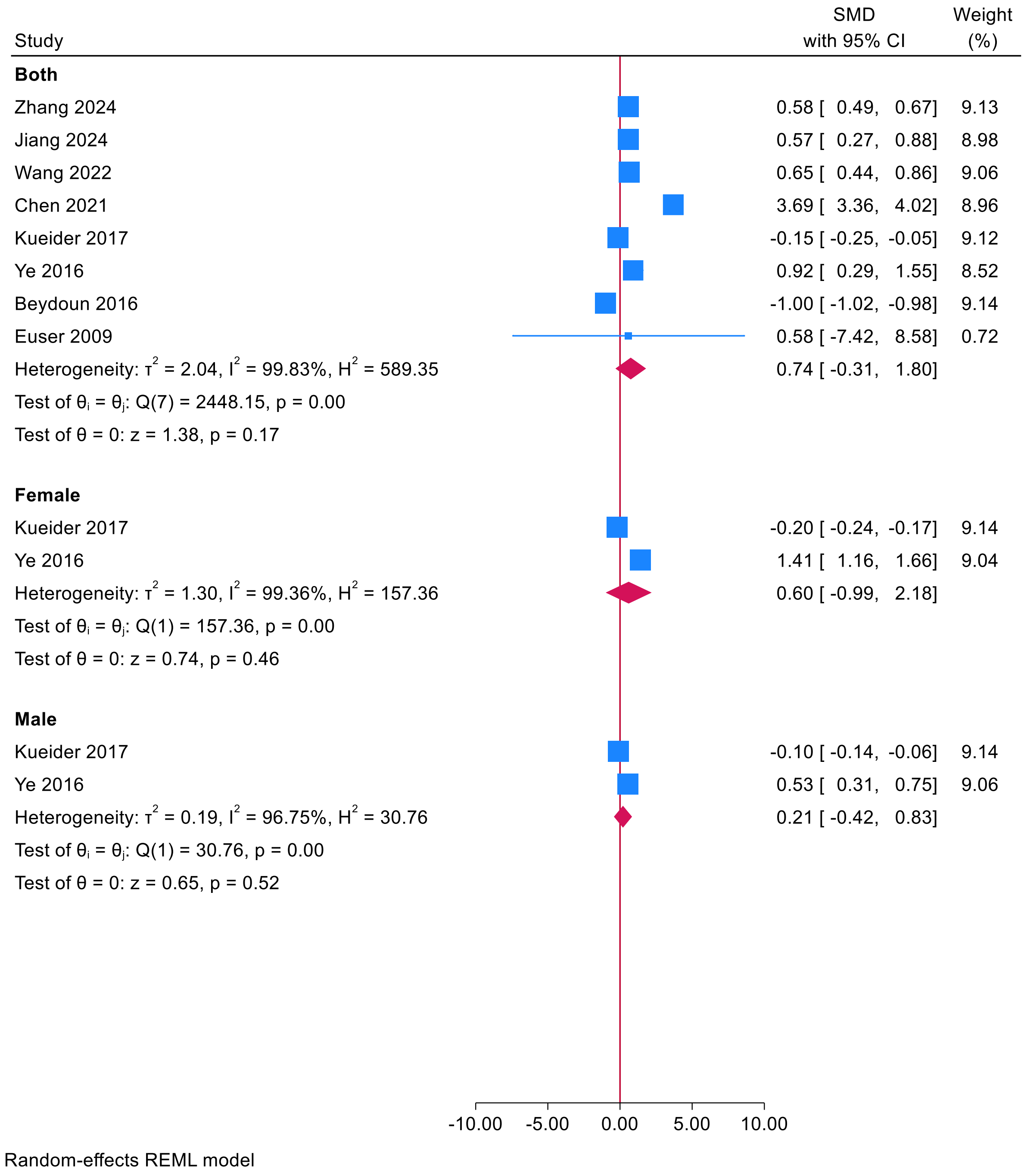

Figure S2: Forest plot for the association between continuous SUA levels and global cognitive performance for prospective cohort studies.

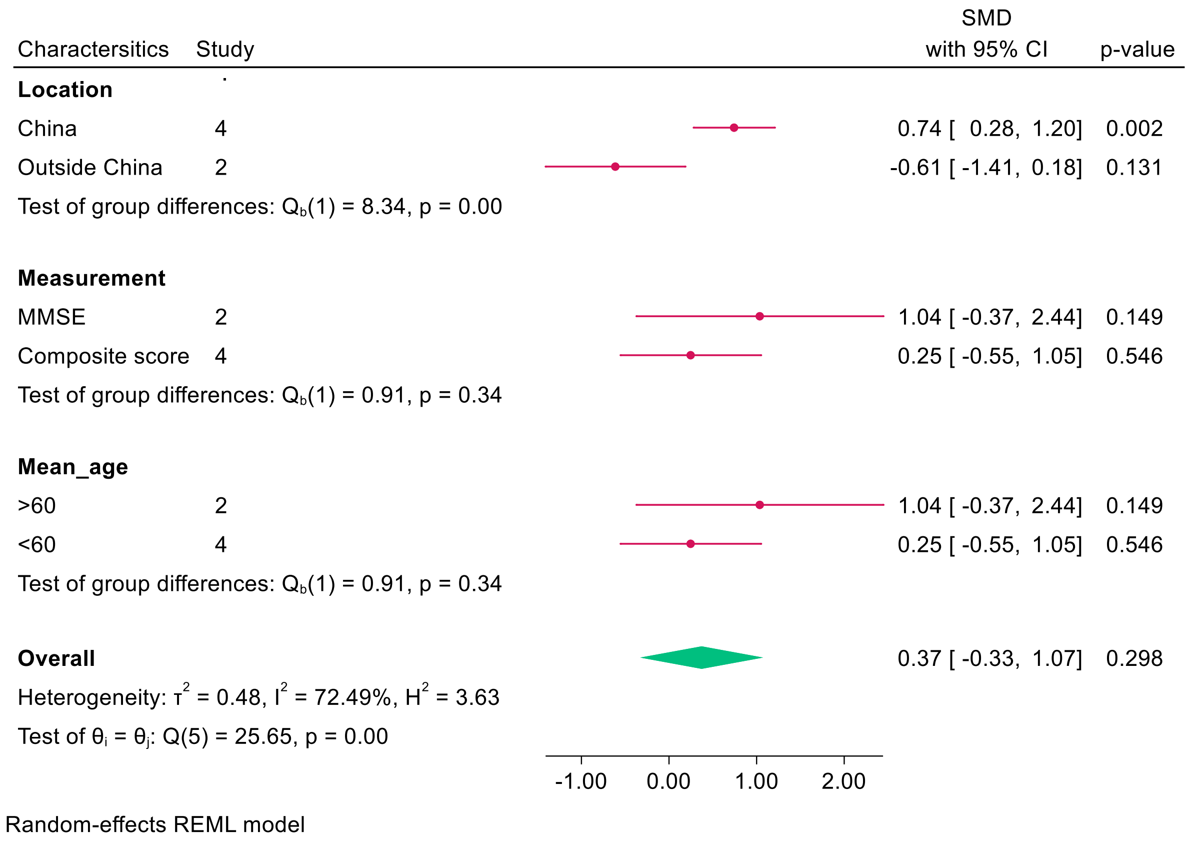

Figure S3: Subgroup analysis for categorical SUA levels (higher vs lower) and global cognitive performance for prospective cohort studies.

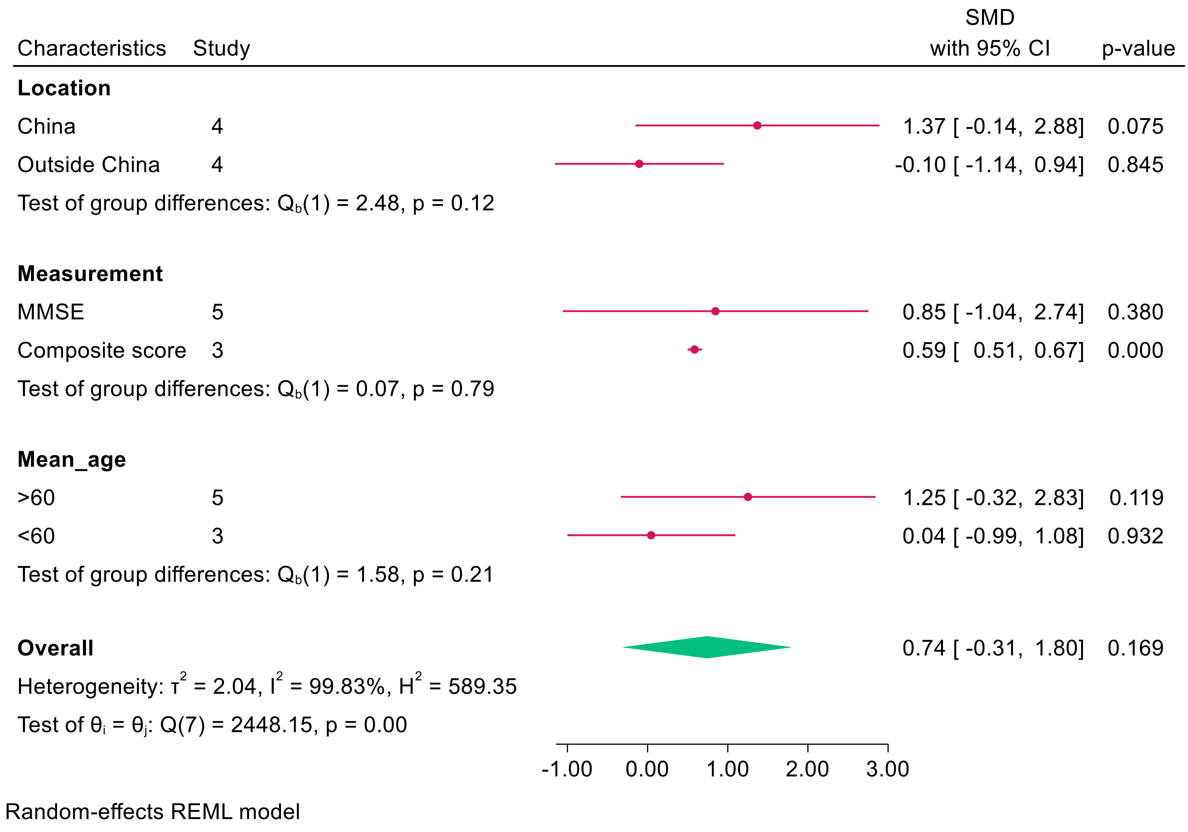

Figure S4: Subgroup analysis for continuous SUA levels and global cognitive performance for prospective cohort studies.

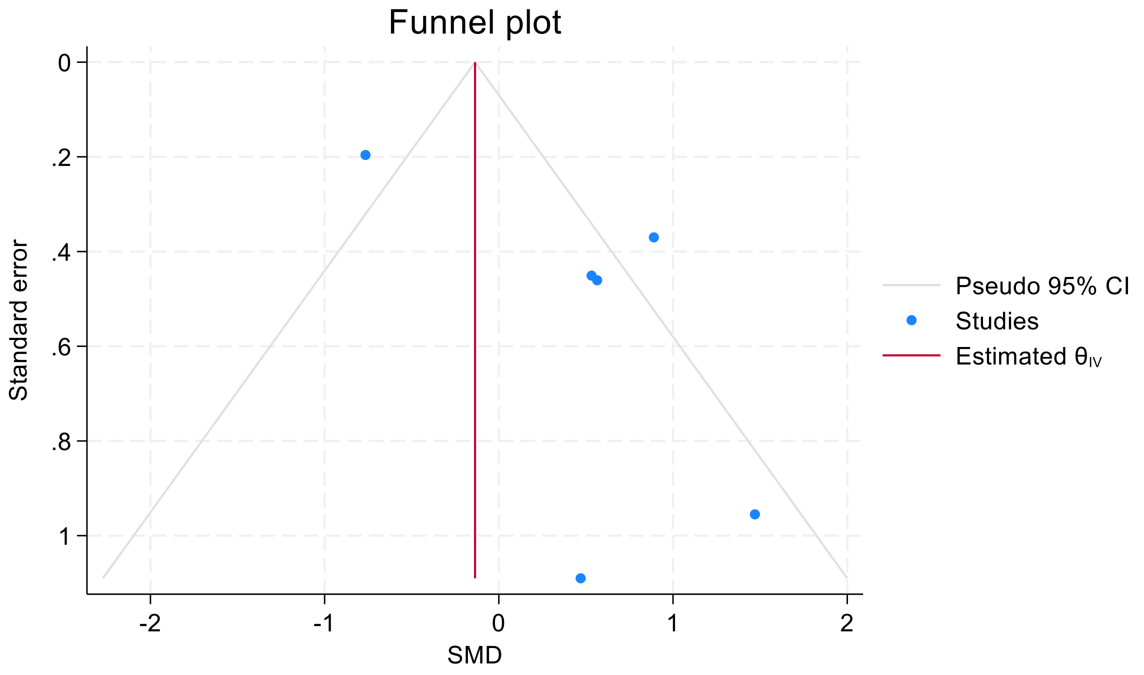

Figure S5: Funnel plot for the association between categorical SUA levels (higher vs lower) and global cognitive performance for prospective cohort study.

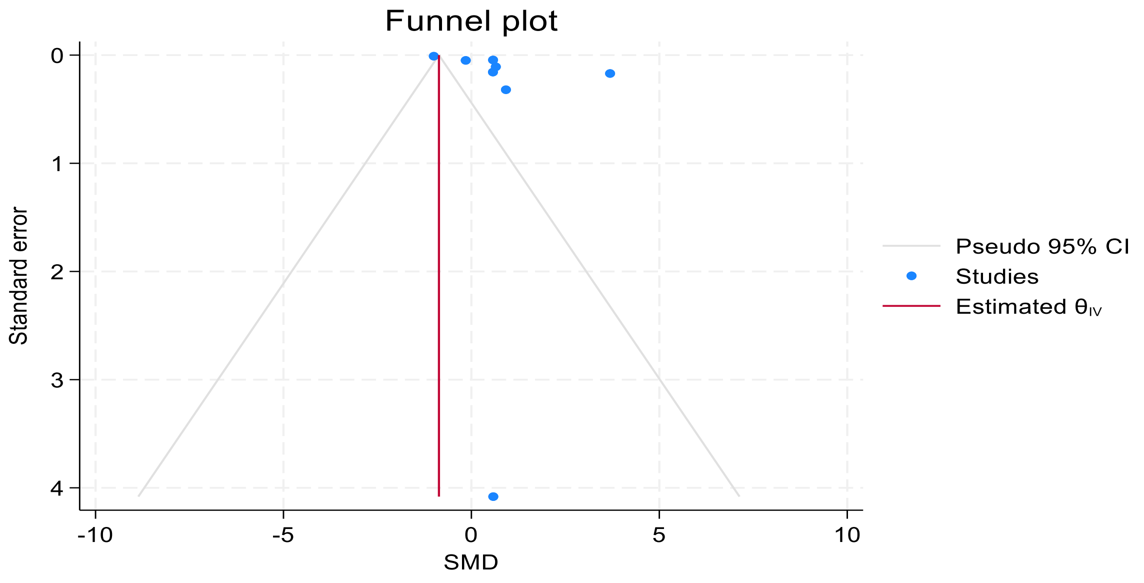

Figure S6: Funnel plot for the association between continuous SUA levels and global cognitive performance for prospective cohort study.

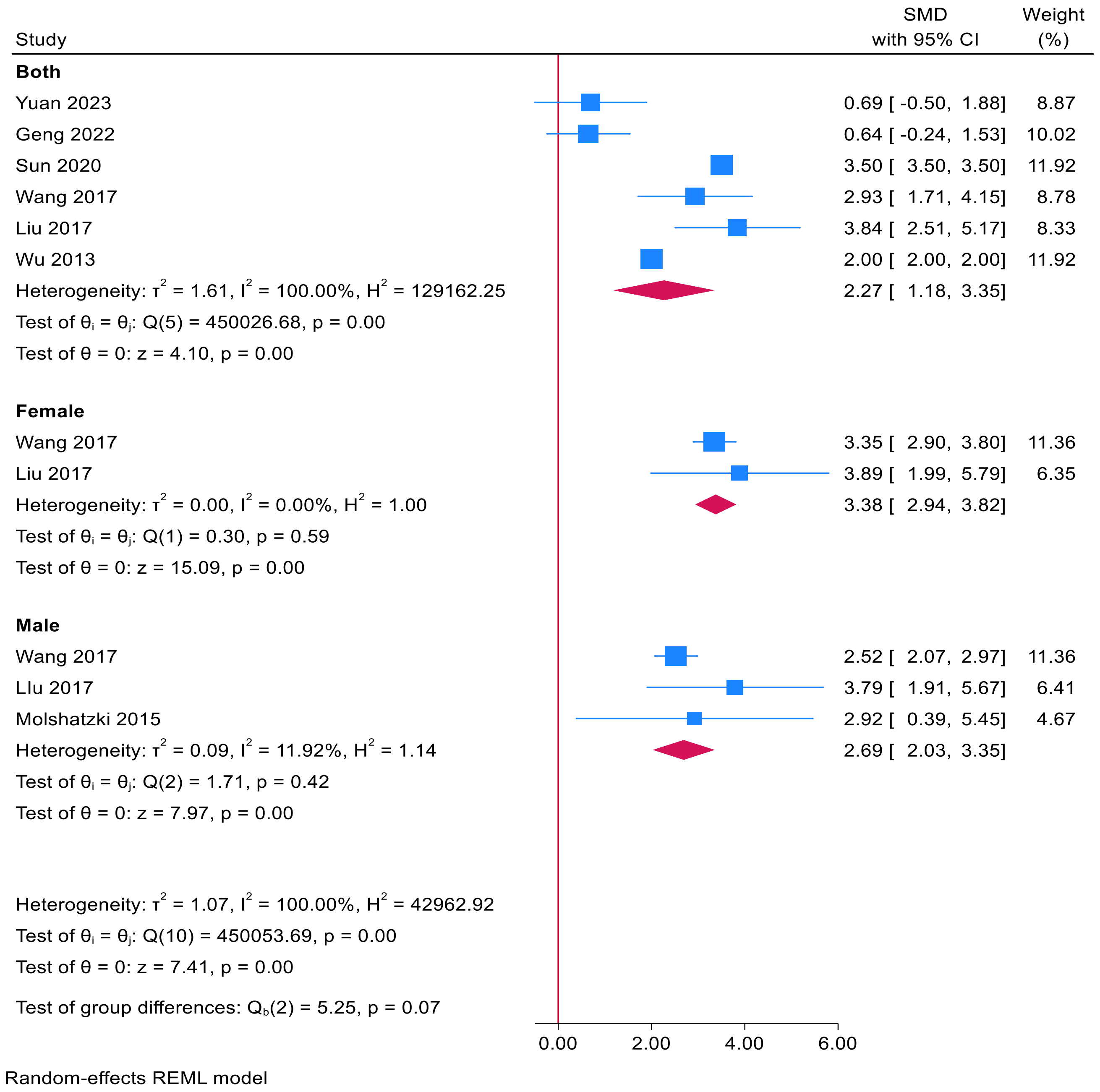

Figure S7: Forest plot for the association between categorical SUA levels (higher vs lower) and global cognitive performance for cross-sectional studies.

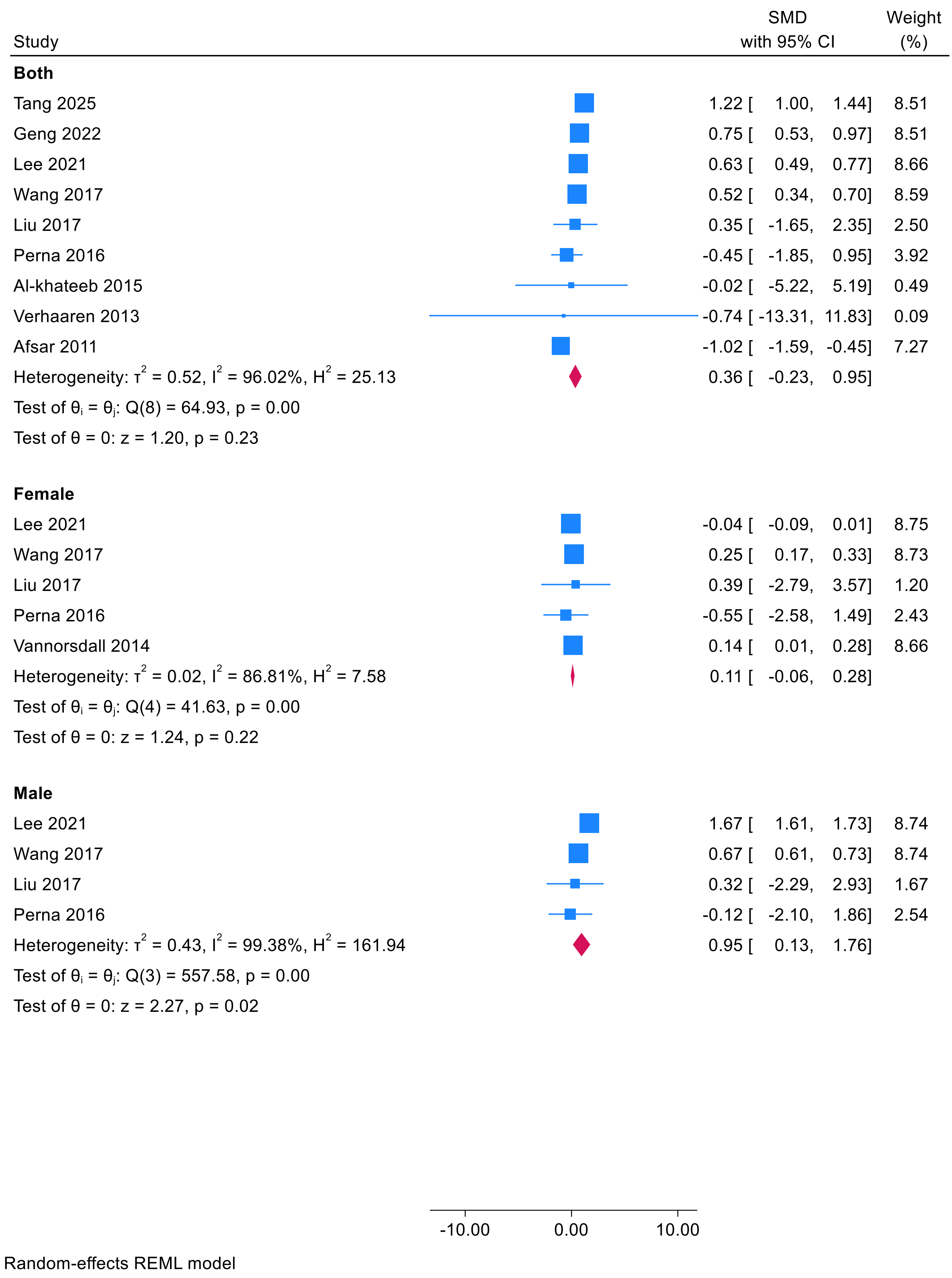

Figure S8: Forest plot for the association between continuous SUA levels and global cognitive performance for cross-sectional studies.

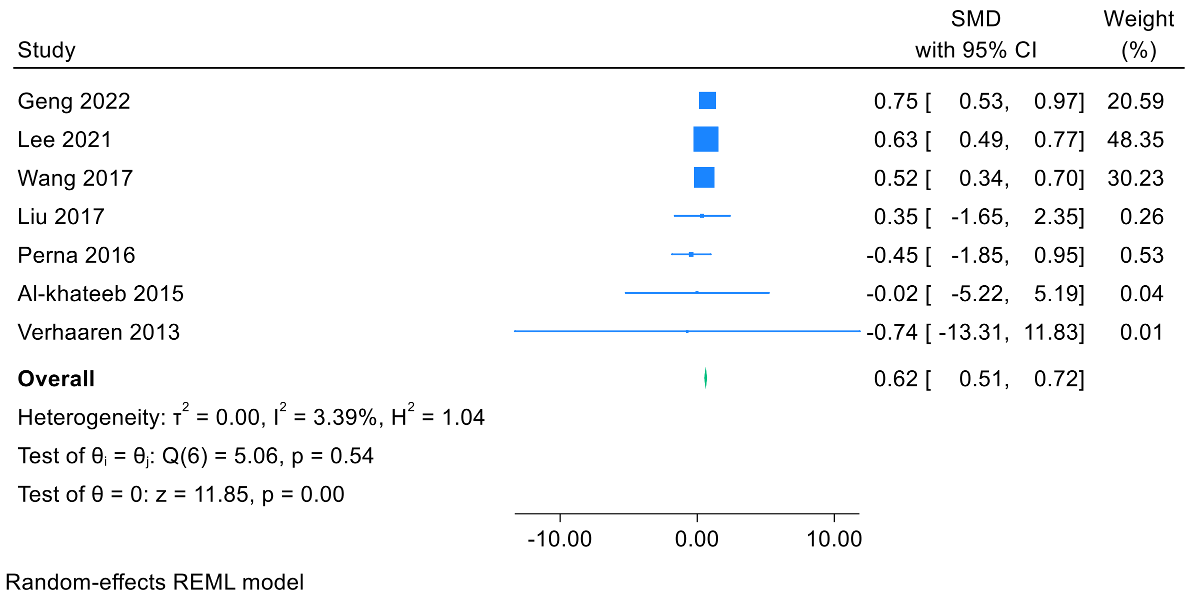

Figure S9: Sensitivity analysis excluding two studies (from both sex; Tang et al. 2025 and Afsar et al.2011, population on ALS and CKD) for cross-section study of continuous SUA level and global cognitive performance.

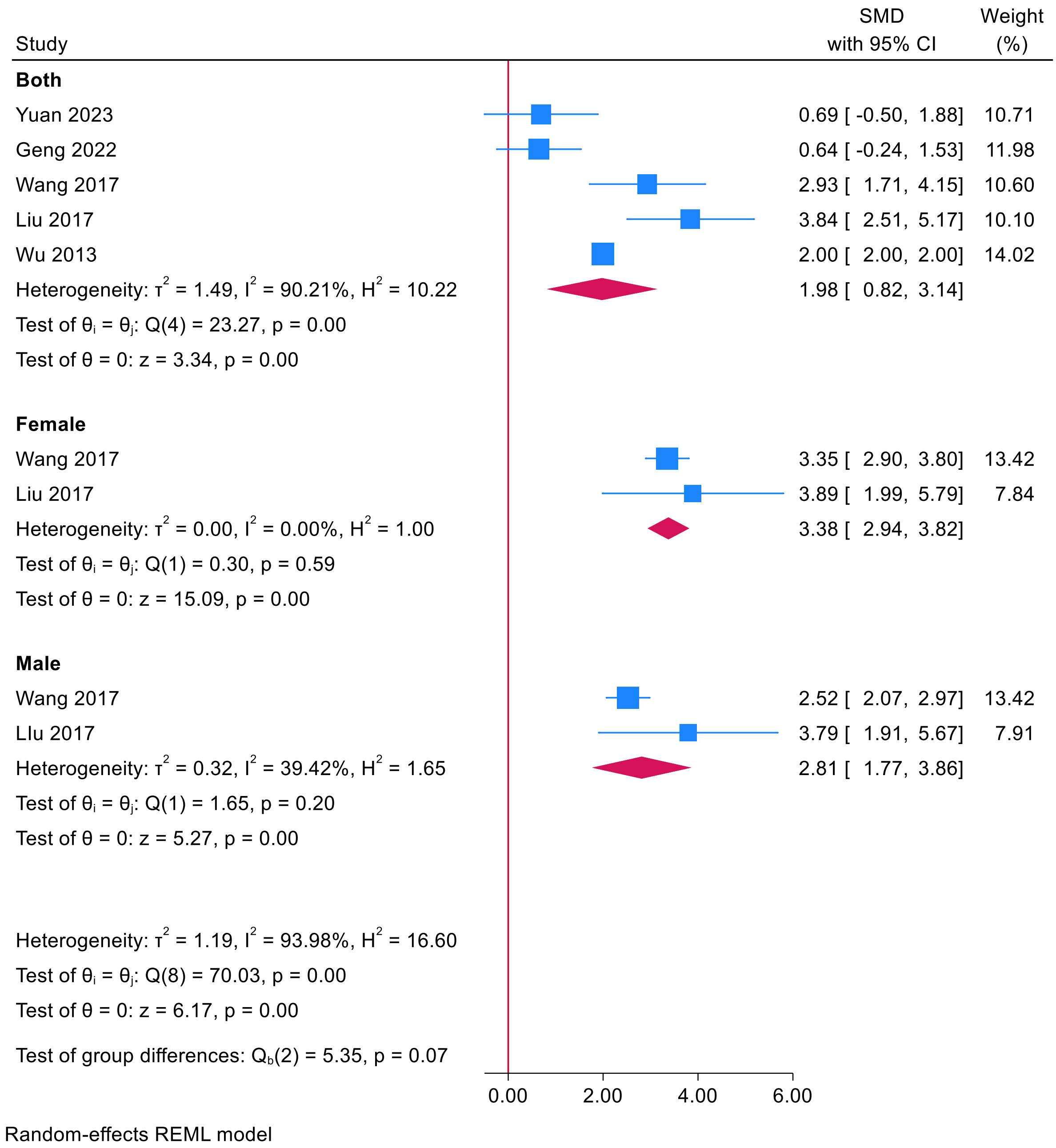

Figure S10: Sensitivity analysis excluding one study from both sex (Sun et al.2020, population on Acute Cerebral Infarction) and one study from male (Molshatzki et al. 2015, population in Myocardial Infarction) for cross-section study for categorical SUA level (higher vs lower) and global cognitive performance.

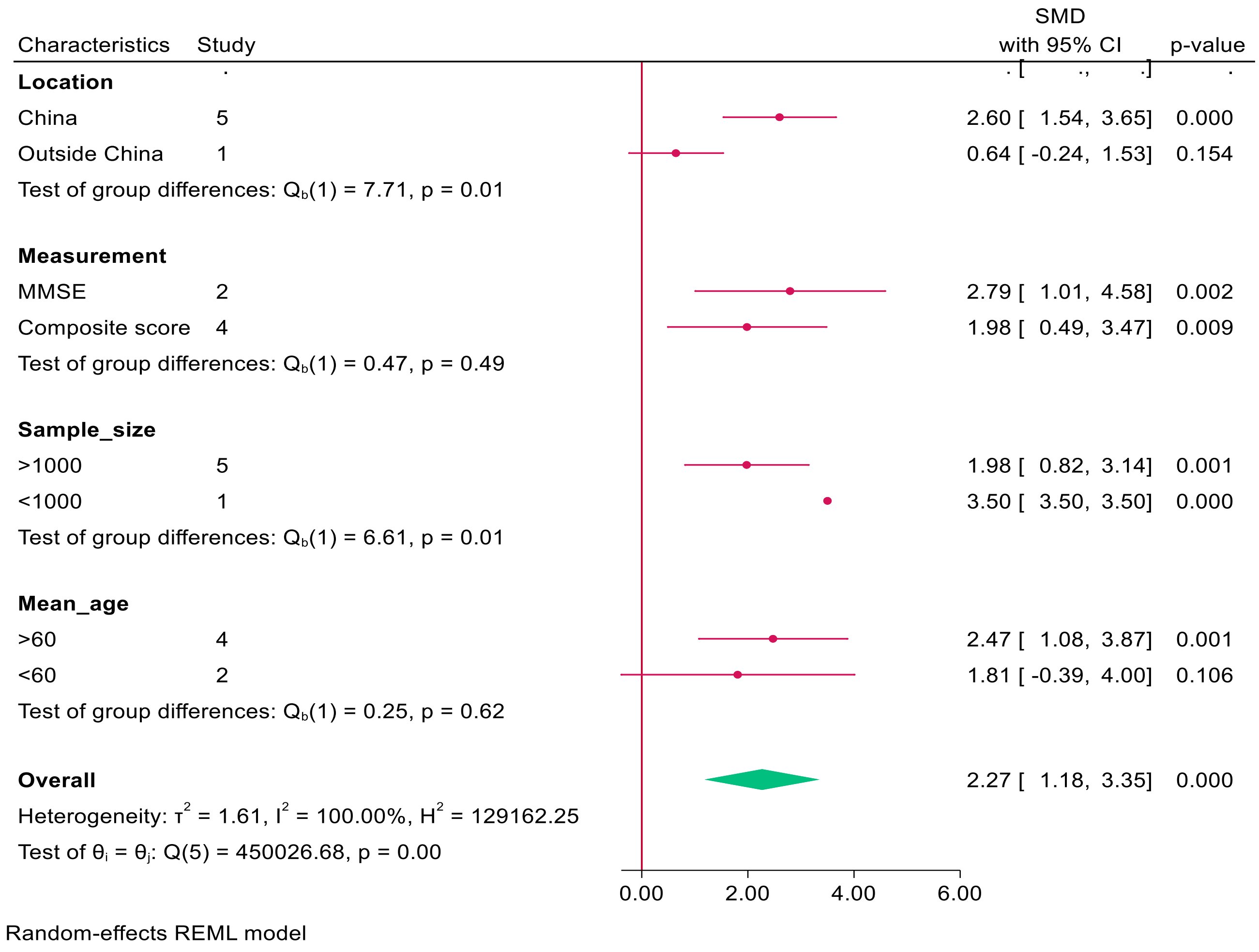

Figure S11: Subgroup analysis for categorical SUA levels (higher vs lower) and global cognitive performance for cross-sectional studies.

Characteristics	Study	SMD with 95% CI	p-value
**Location**			
China	3	0.83 [0.22, 1.43]	0.007
Outside China	7	0.09 [-0.56, 0.74]	0.778
Test of group differences: $Q_b(1) = 2.63$, p = 0.10			
**Measurement**			
MMSE	5	0.00 [-0.77, 0.77]	0.996
Composite score	4	0.60 [0.37, 0.83]	0.000
ECAS	1	1.22 [1.00, 1.44]	0.000
Test of group differences: $Q_b(2) = 20.22$, p = 0.00			
**Sample size**			
>1000	5	0.62 [0.51, 0.72]	0.000
<1000	5	0.12 [-1.04, 1.28]	0.833
Test of group differences: $Q_b(1) = 0.68$, p = 0.41			
**Mean age**			
>60	8	0.12 [-0.48, 0.71]	0.705
<60	2	0.87 [0.18, 1.55]	0.013
Test of group differences: $Q_b(1) = 2.62$, p = 0.11			

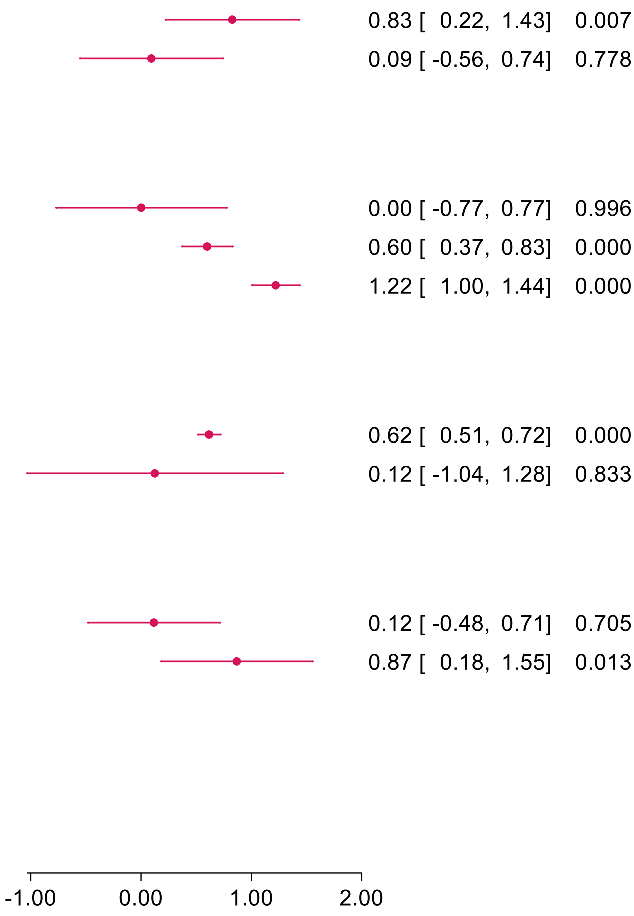

Random-effects REML model

Figure S12: Subgroup analysis for continuous SUA levels and global cognitive performance for cross-sectional studies.

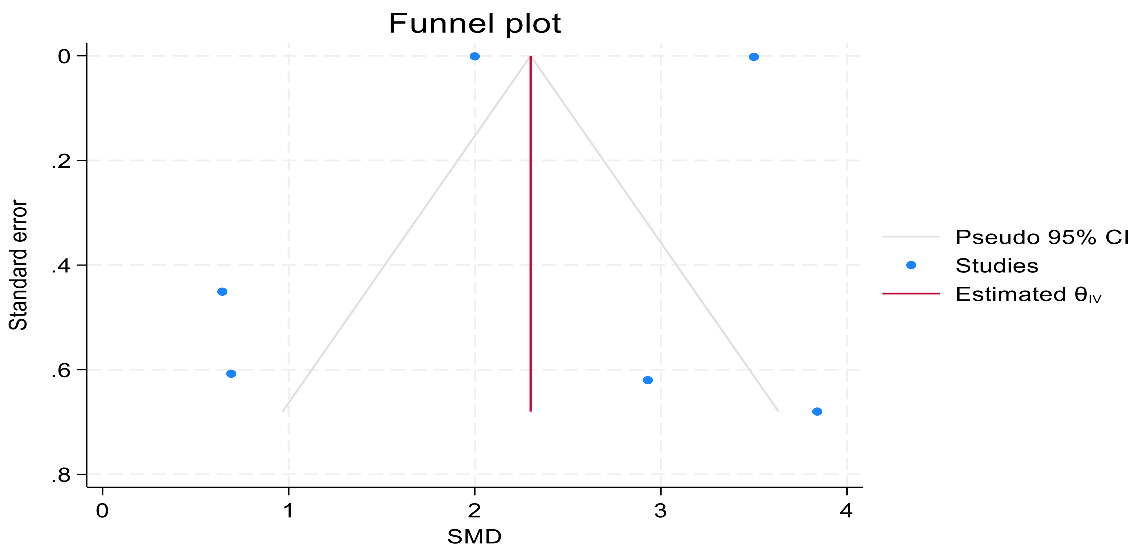

Figure S13: Funnel plot for the association between categorical SUA levels (higher vs lower) and global cognitive performance for cross-sectional study.

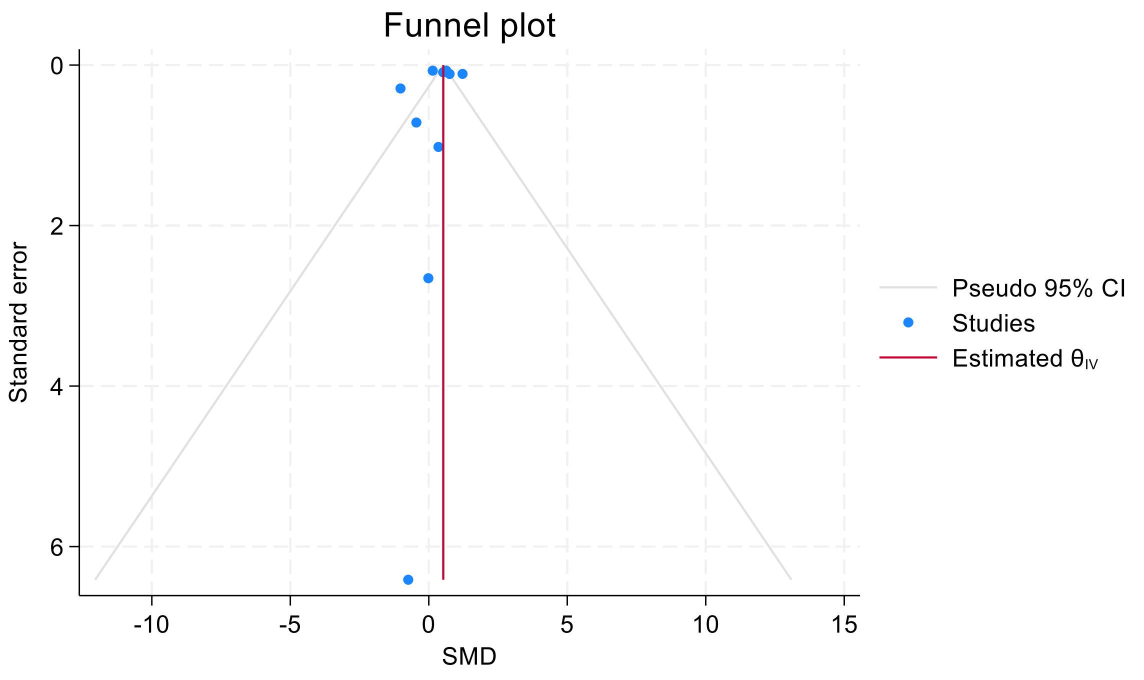

Figure S14: Funnel plot for the association between continuous SUA levels and global cognitive performance for cross-sectional study.

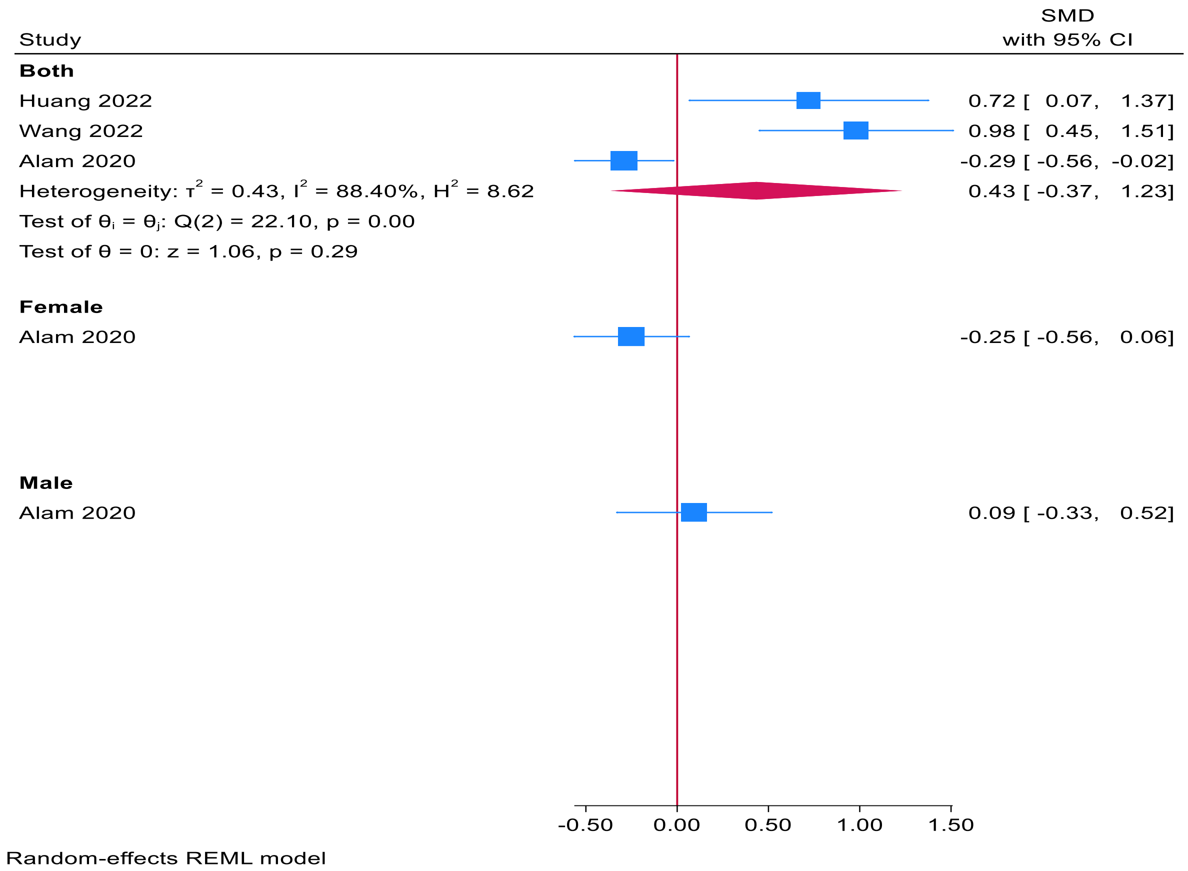

Figure S15: Forest plot for the association between categorical SUA levels (higher vs lower) and executive function for prospective cohort studies.

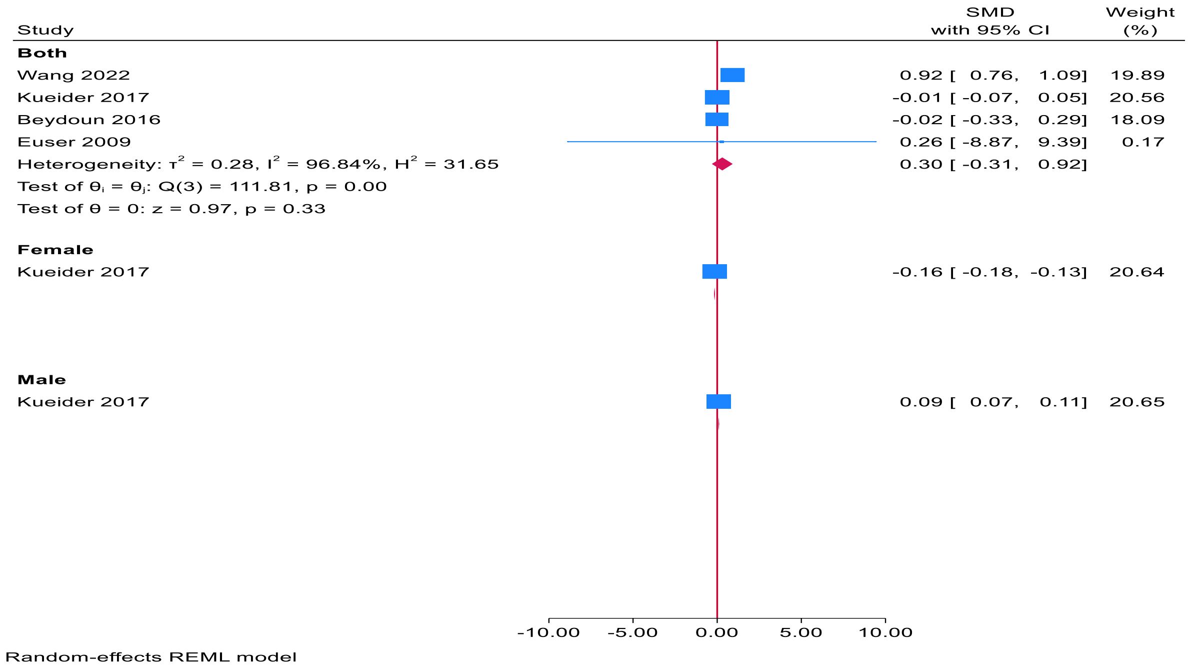

Figure S16: Forest plot for the association between continuous SUA levels and executive function for prospective cohort studies.

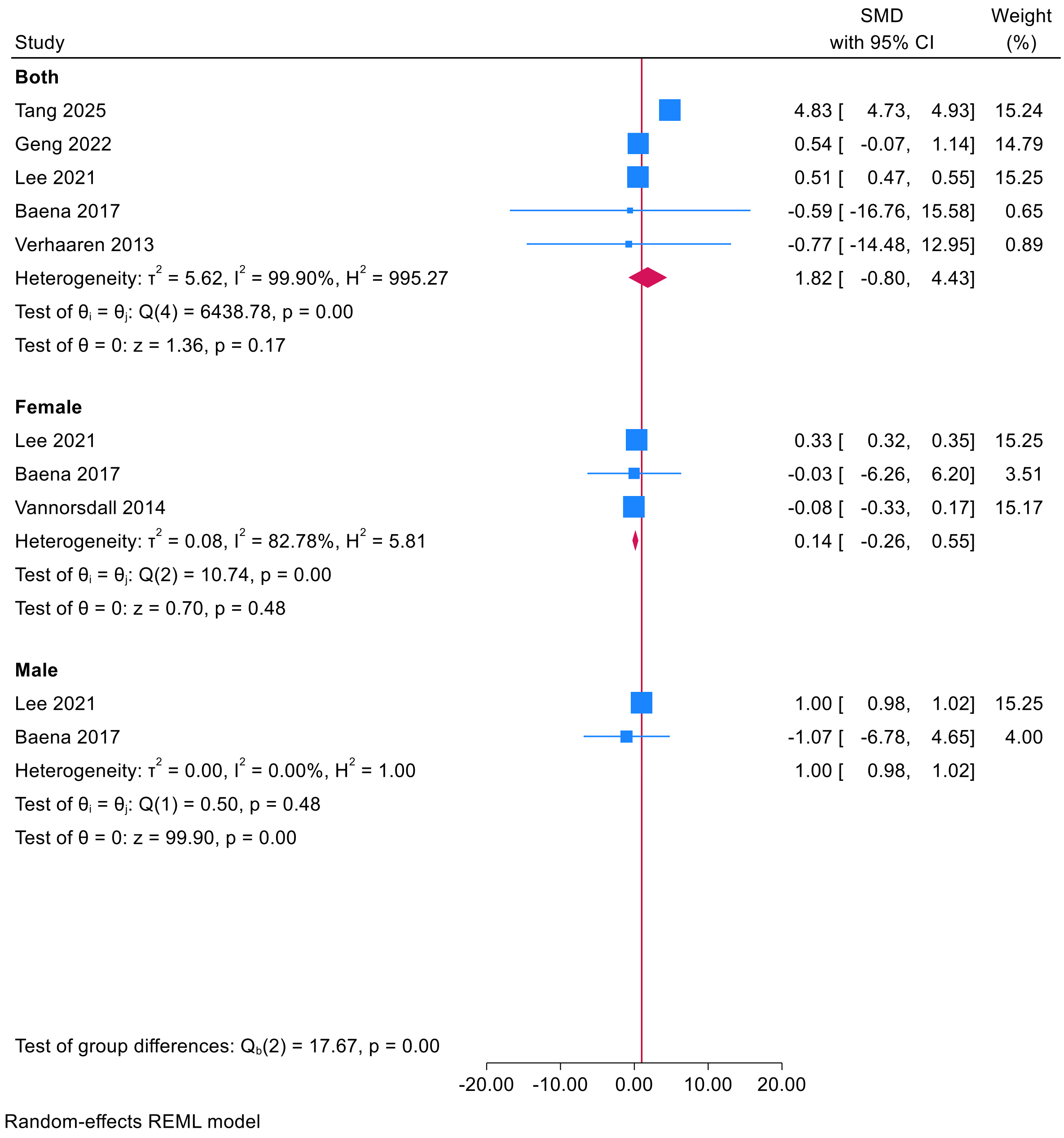

Figure S17A: Forest plot for the association between continuous SUA levels and executive function for cross-sectional studies.

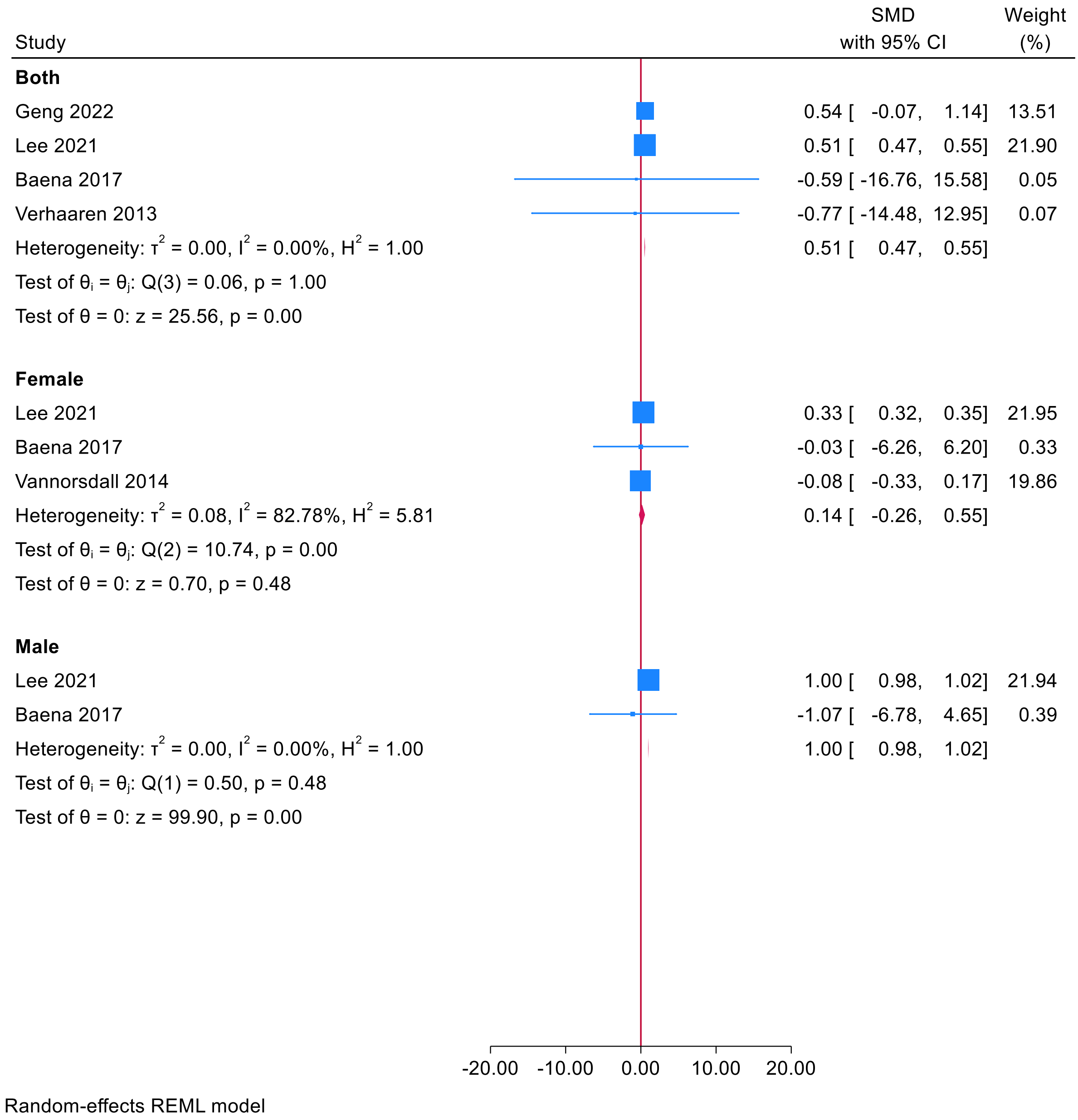

Figure S17B: Sensitivity analysis excluding a study (Tang et al. 2025; population on ALS) for cross-section study of continuous SUA level and executive function.

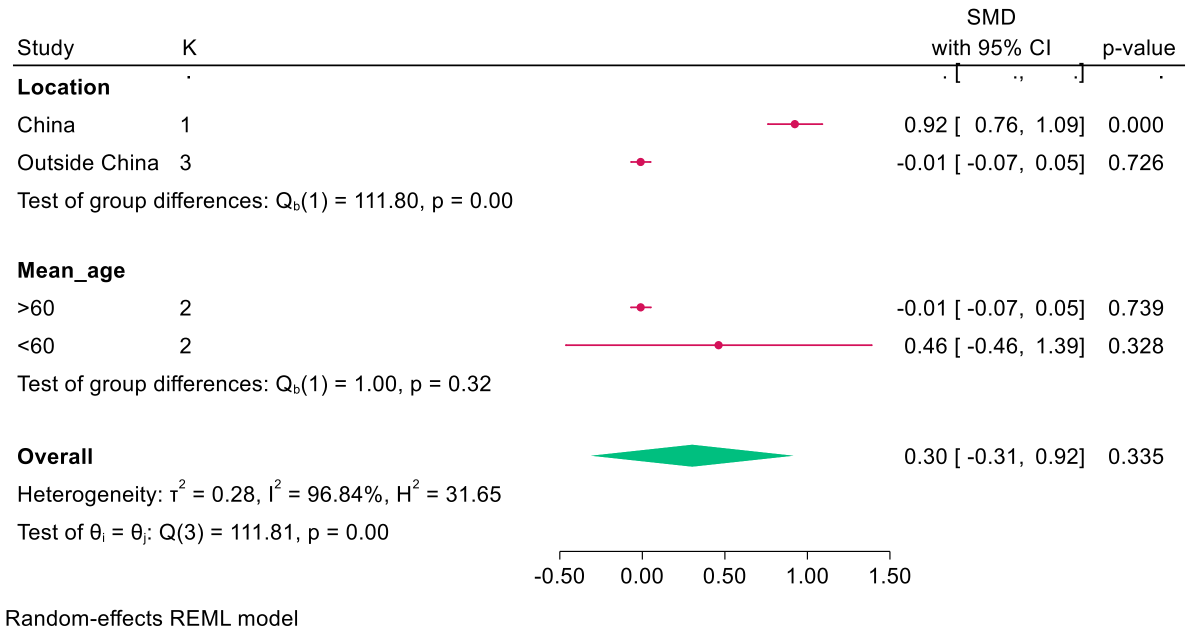

Figure S18: Subgroup analysis for continuous SUA levels and executive function for prospective cohort studies.

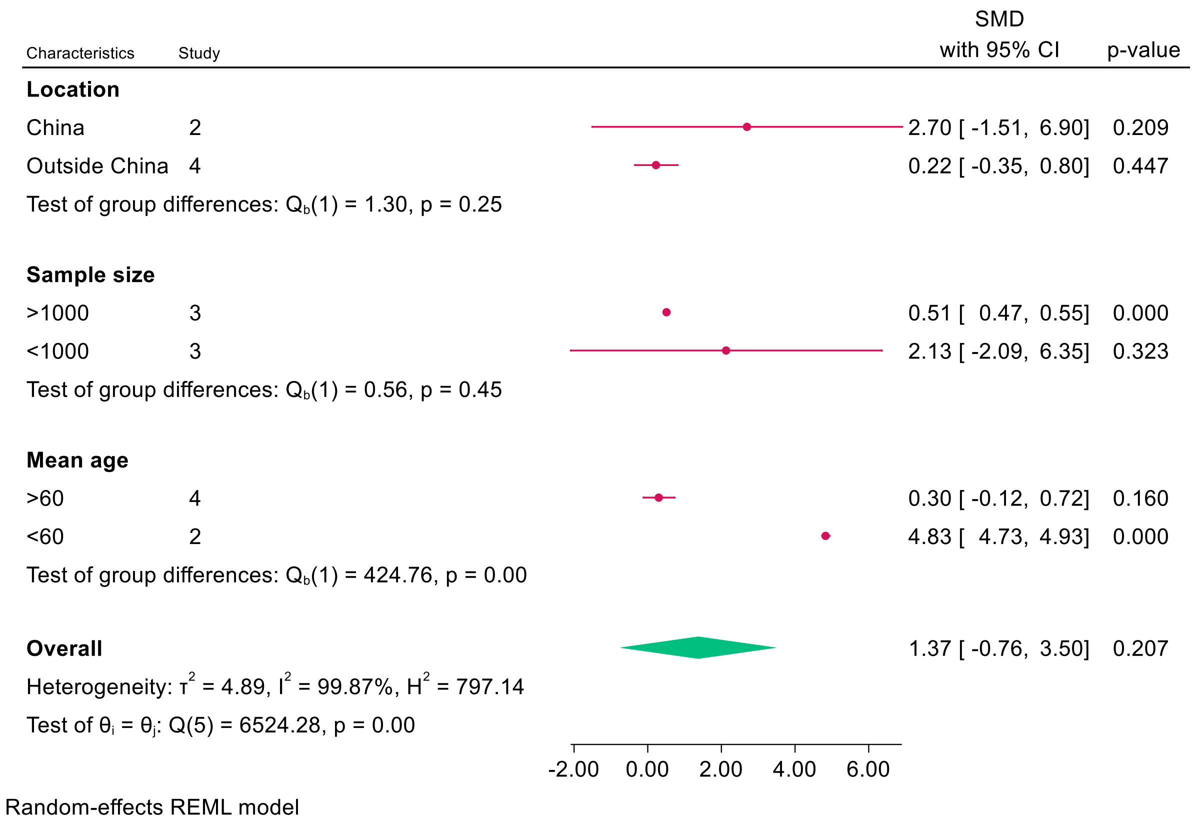

Figure S19: Subgroup analysis for continuous SUA levels and executive function for cross-sectional studies.

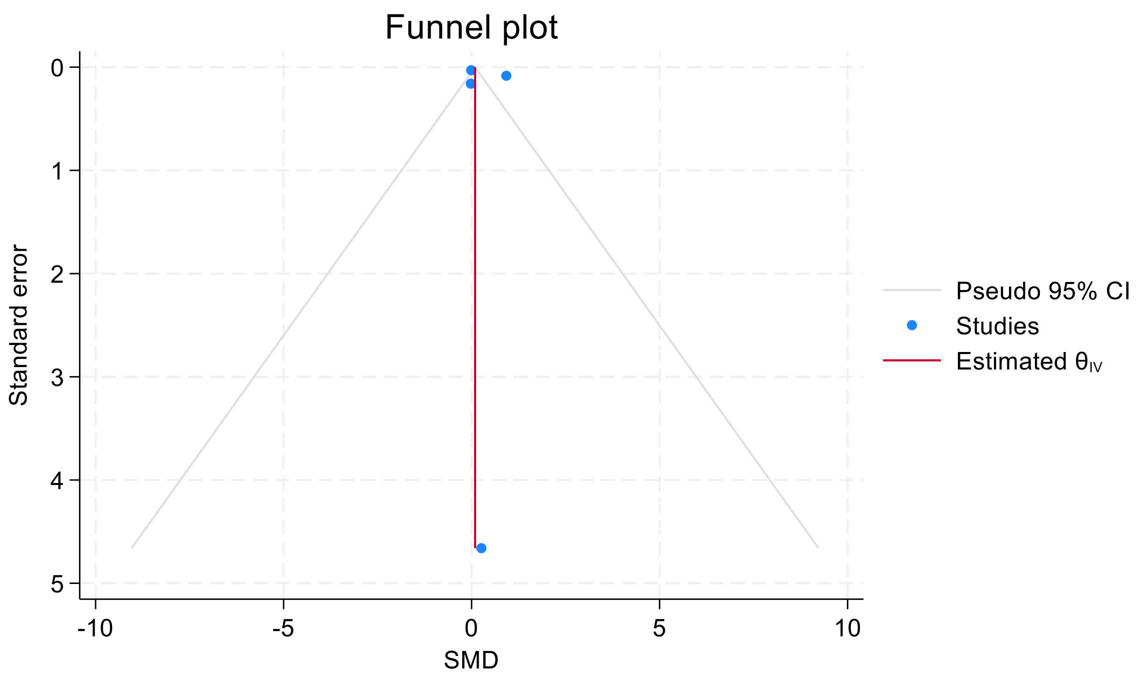

Figure S20: Funnel plot for the association between continuous SUA levels and executive function for prospective cohort study.

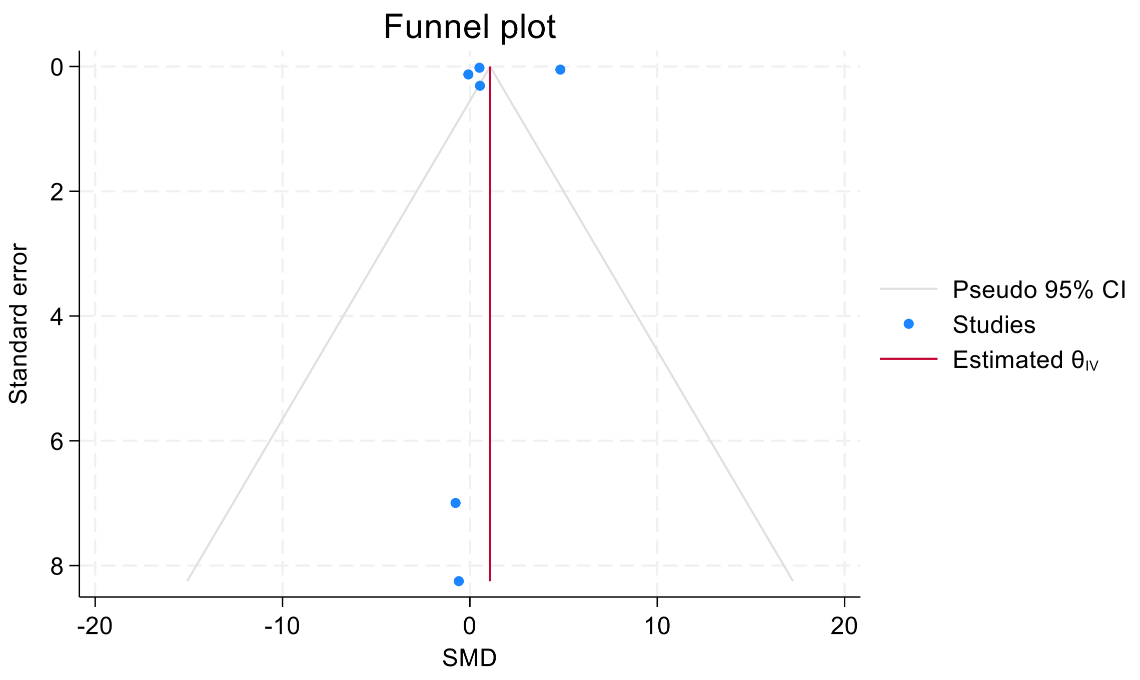

Figure S21: Funnel plot for the association between continuous SUA levels and executive function for cross-sectional study.

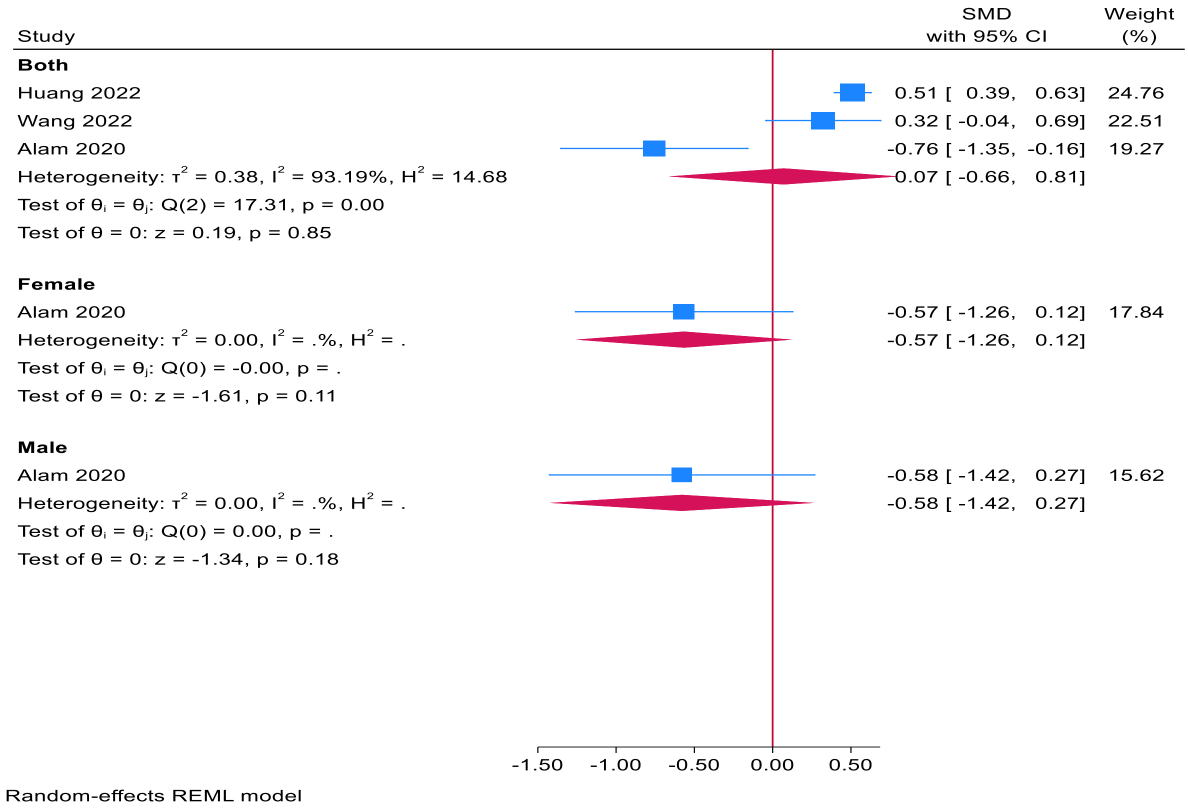

Figure S22: Forest plot for the association between categorical SUA levels (higher vs lower) and learning and memory for prospective cohort studies.

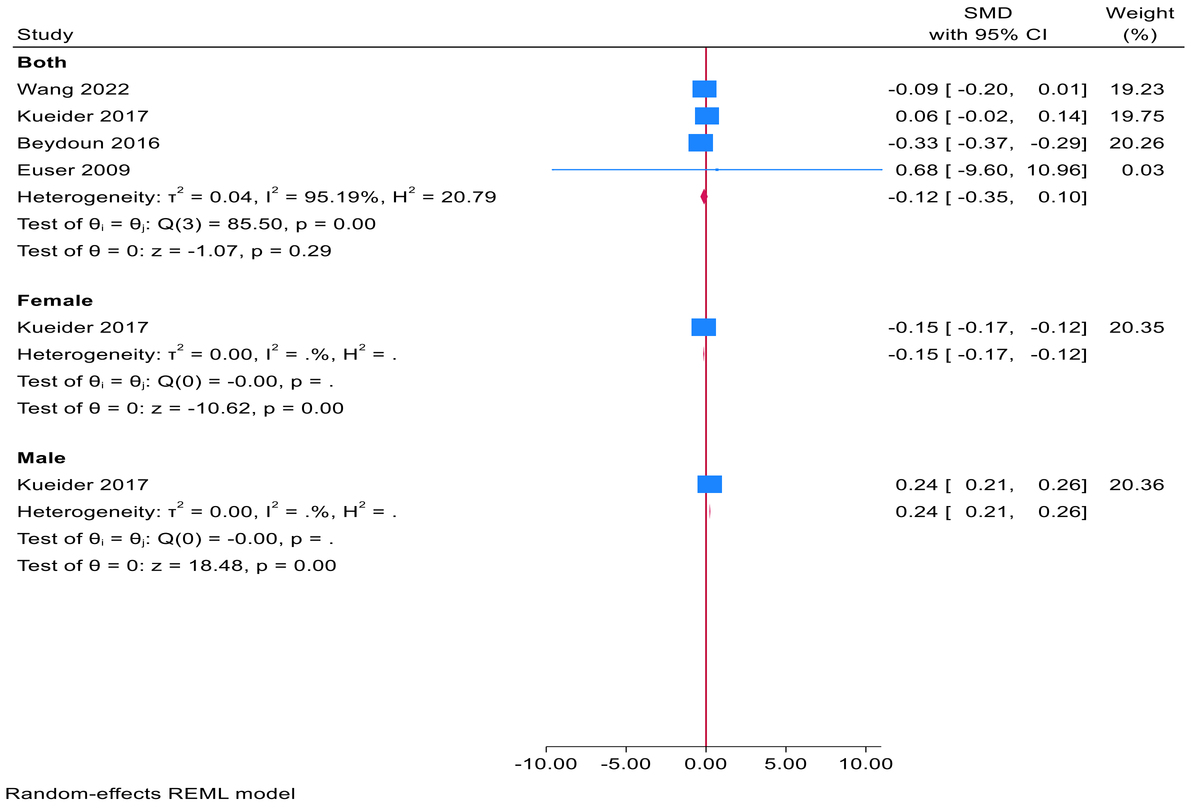

Figure S23: Forest plot for the association between continuous SUA levels and learning and memory for prospective cohort studies.

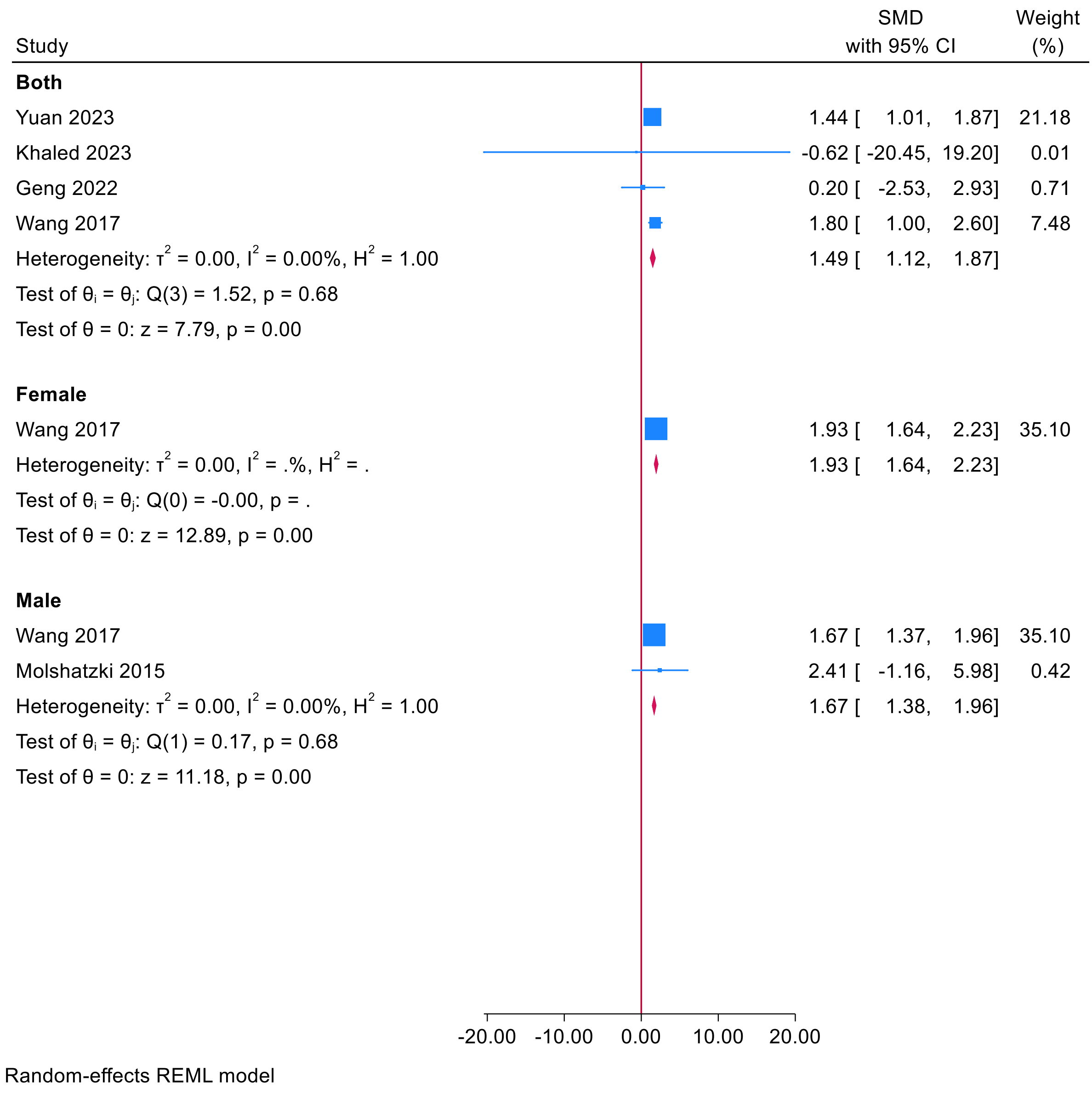

Figure S24: Forest plot for the association between categorical SUA levels (higher vs lower) and learning and memory for cross-sectional studies.

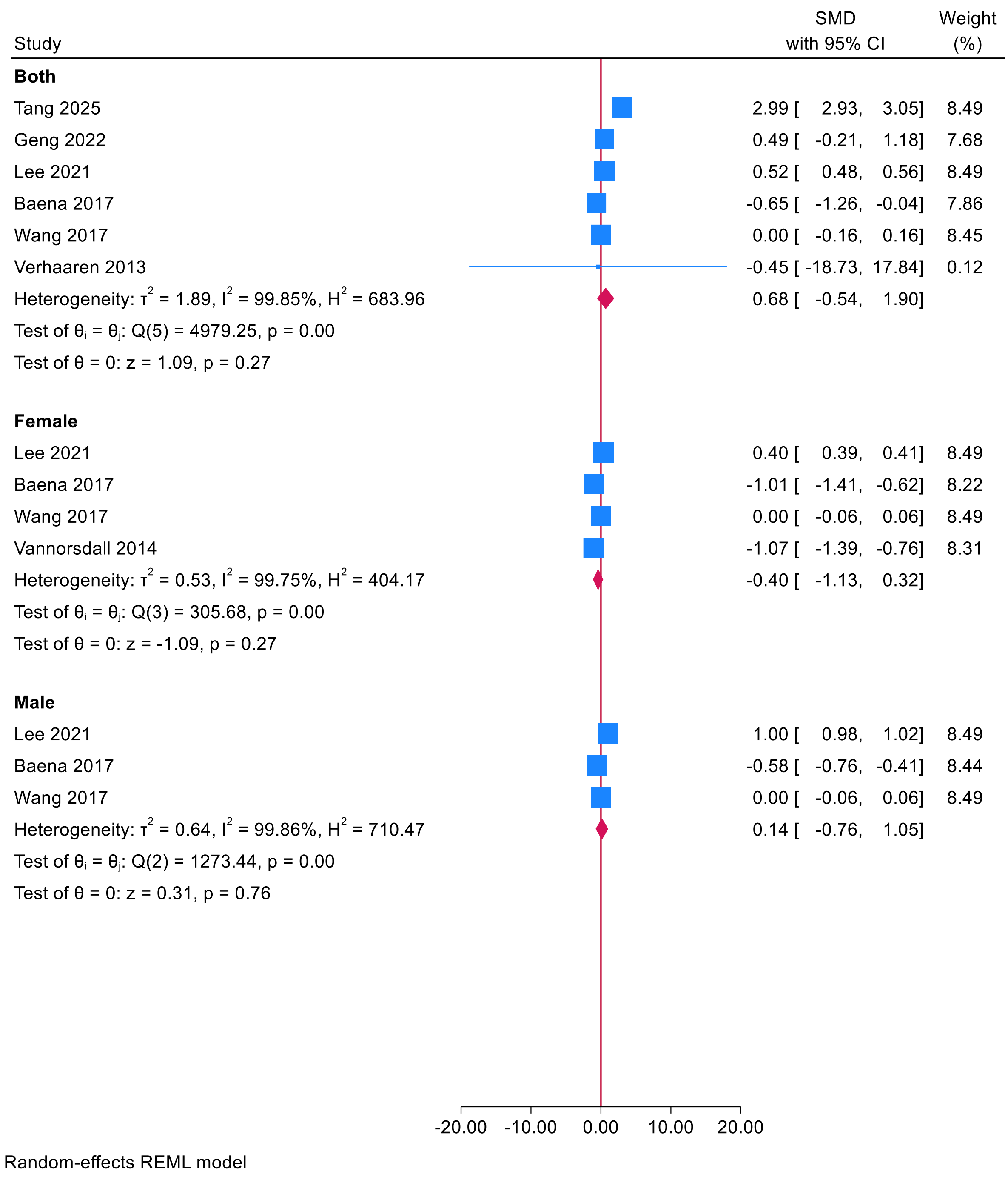

Figure S25: Forest plot for the association between continuous SUA levels and learning and memory for cross-sectional studies.

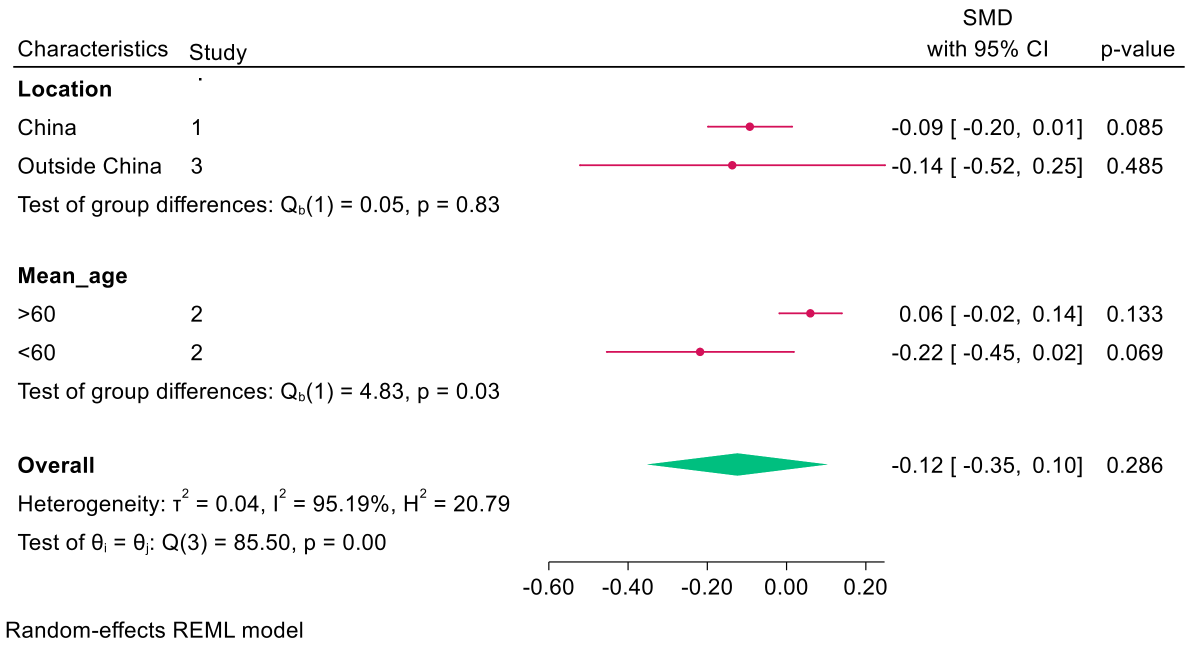

Figure S26: Subgroup analysis for continuous SUA levels and learning and memory for prospective cohort studies.

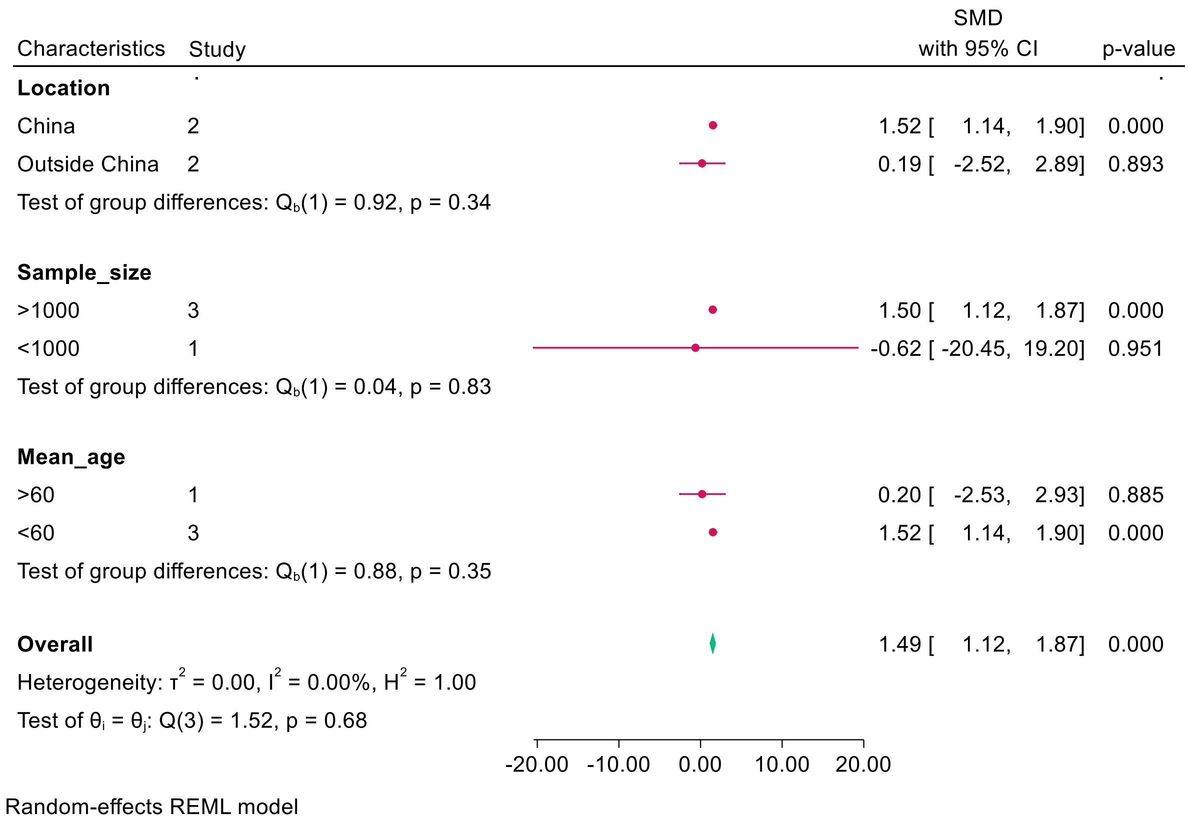

Figure S27: Subgroup analysis for categorical SUA levels (higher vs lower) and learning and memory for cross-sectional studies.

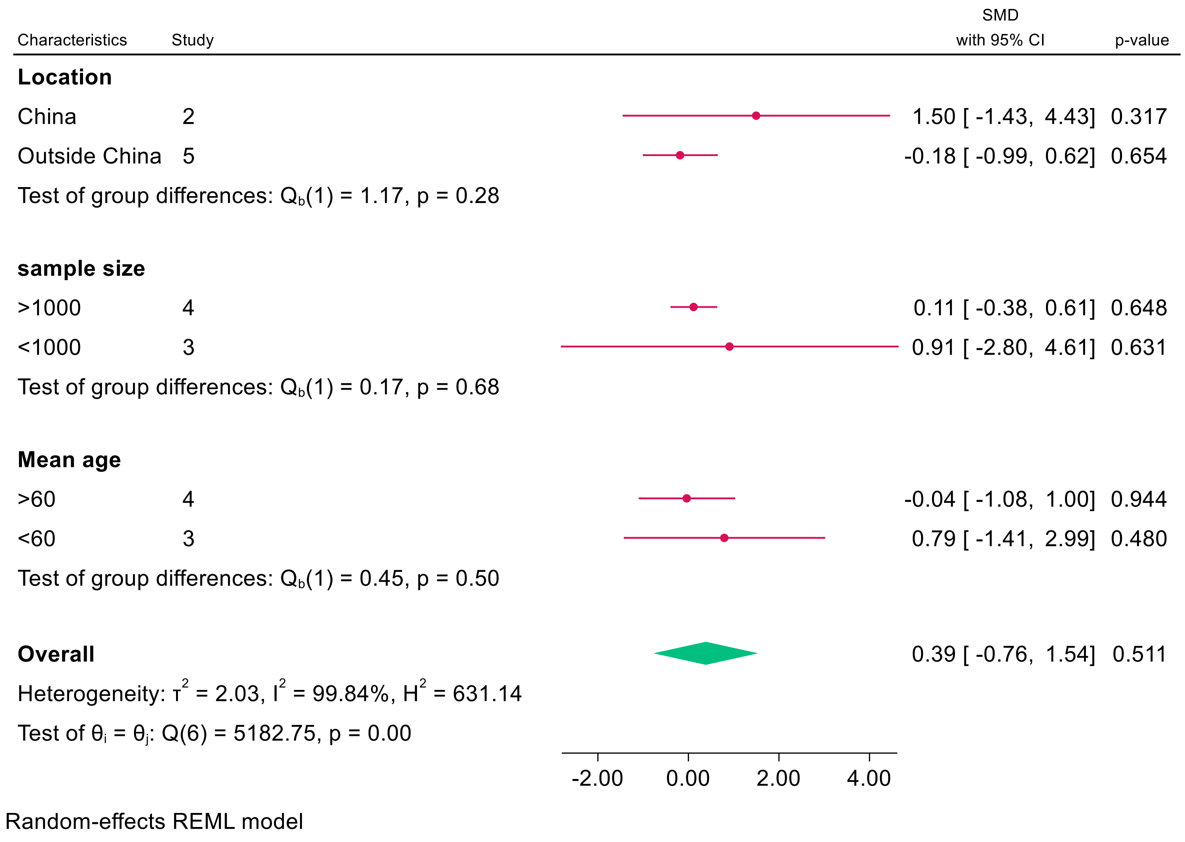

Figure S28: Subgroup analysis for continuous SUA levels and learning and memory for cross-sectional studies.

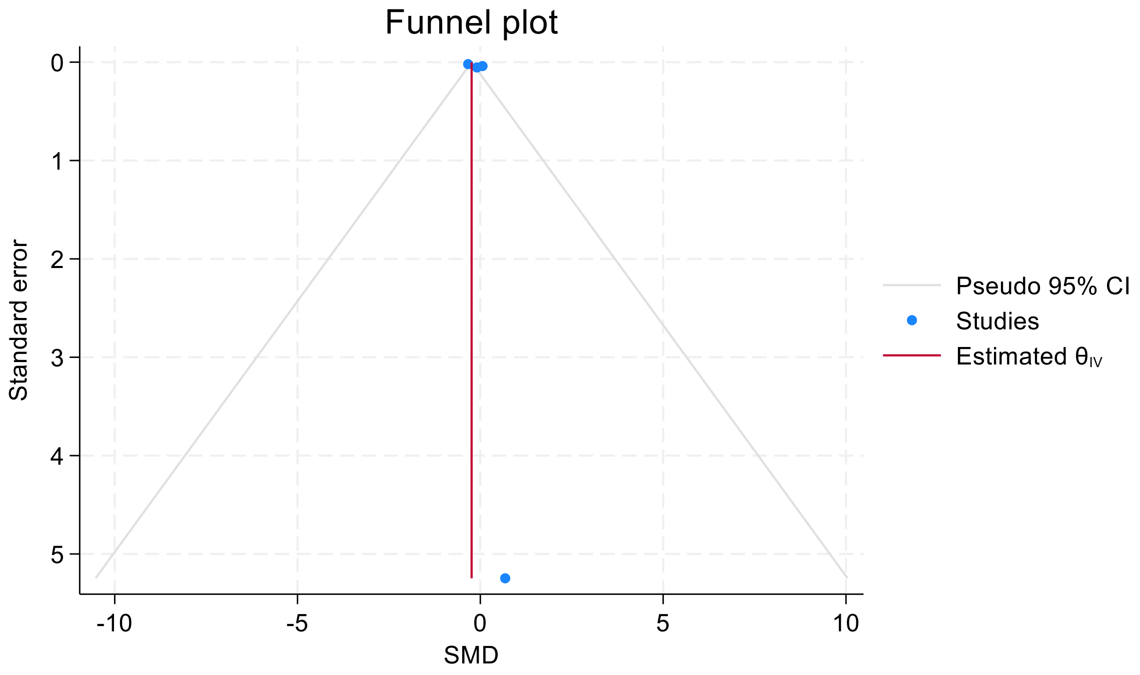

Figure S29: Funnel plot for the association between continuous SUA levels and learning and memory for prospective cohort study.

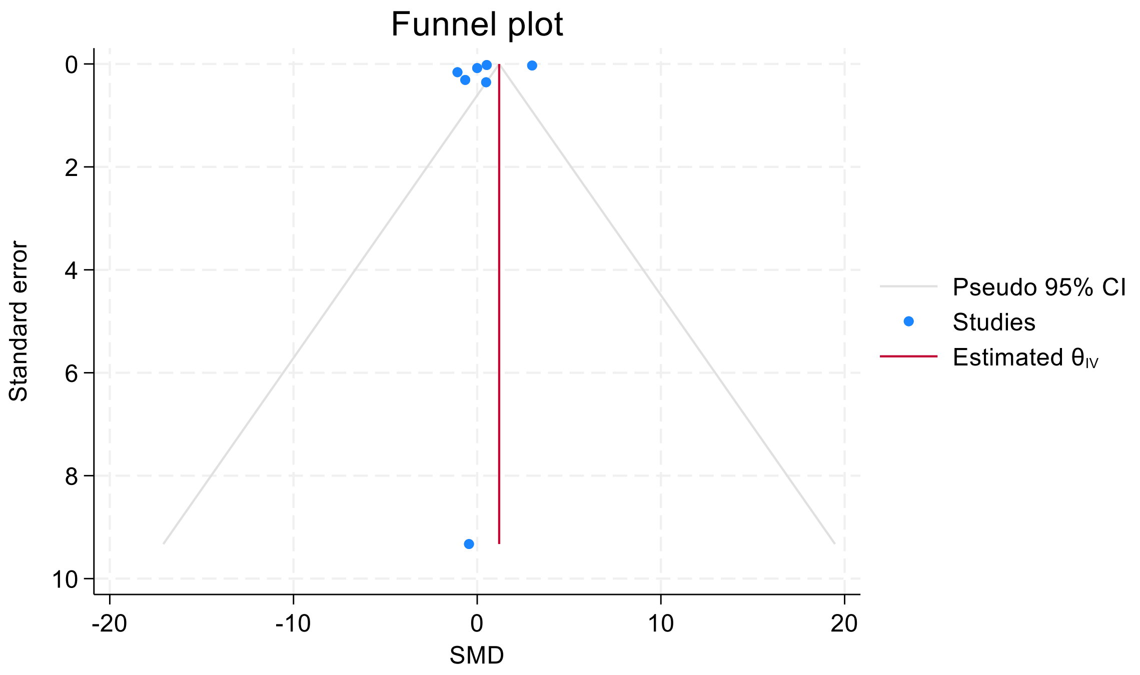

Figure S30: Funnel plot for the association between continuous SUA levels and learning and memory for cross-sectional study.

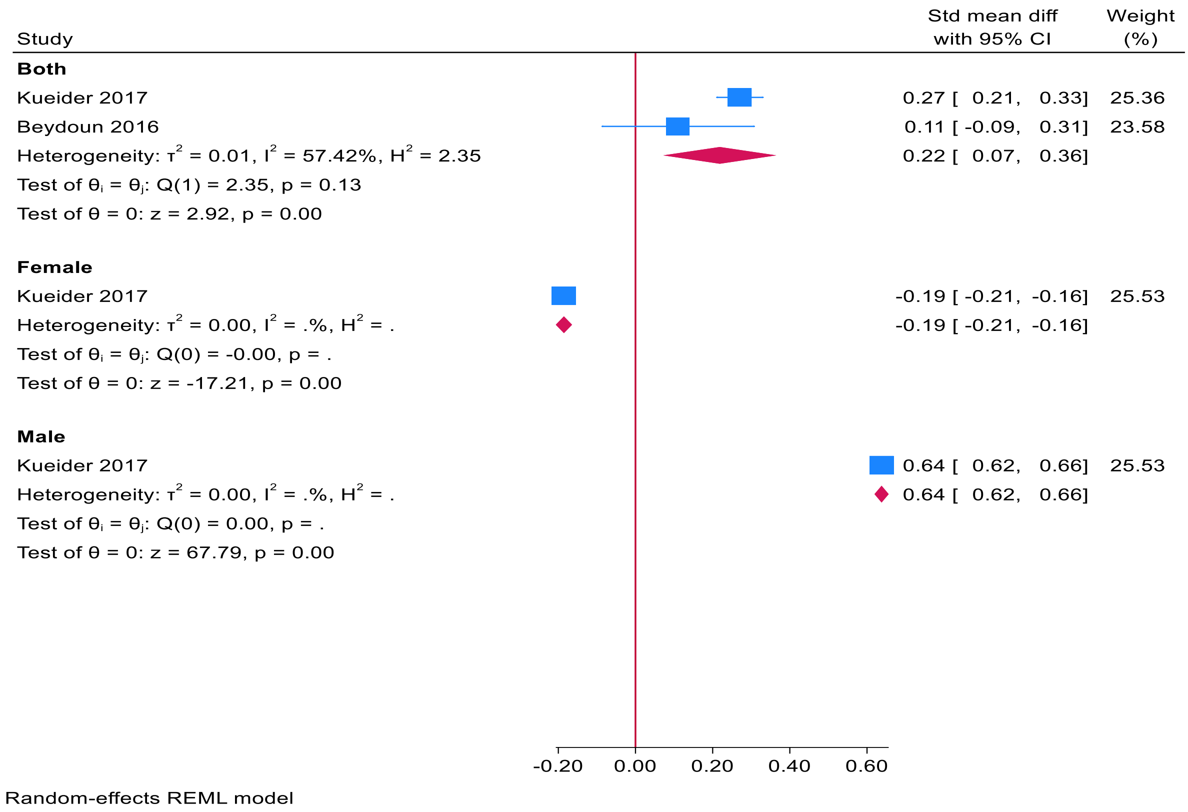

Figure S31: Forest plot for the association between continuous SUA levels and attention for prospective cohort studies.

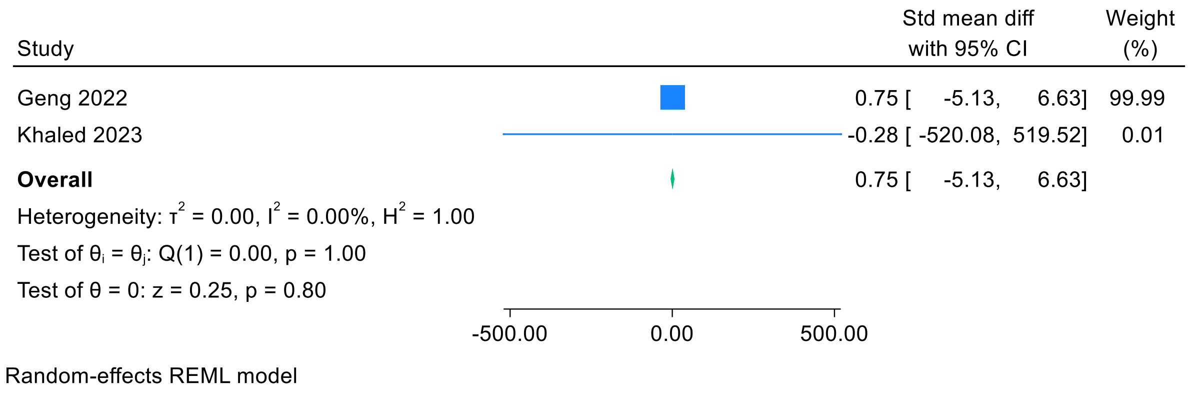

Figure S32: Forest plot for the association between categorical SUA levels (higher vs lower) and attention for cross-sectional studies.

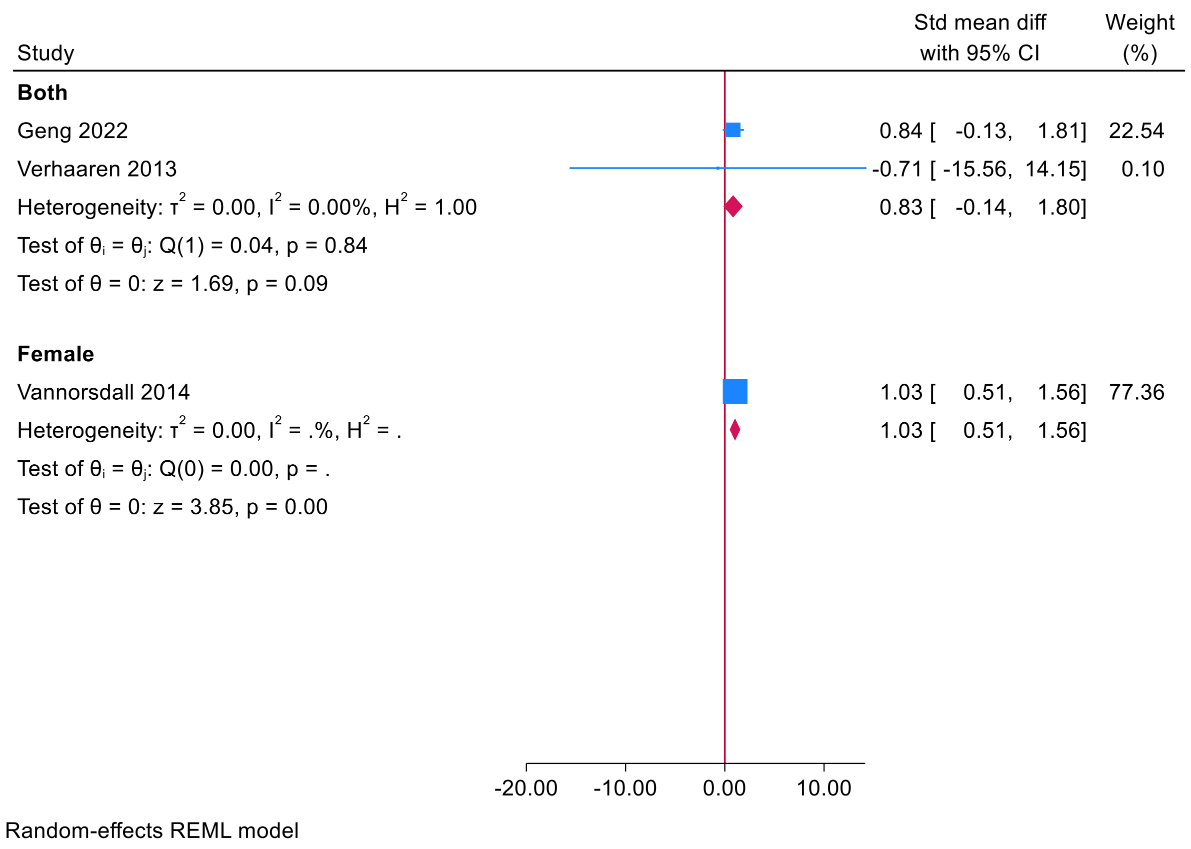

Figure S33: Forest plot for the association between continuous SUA levels and attention for cross-sectional studies.

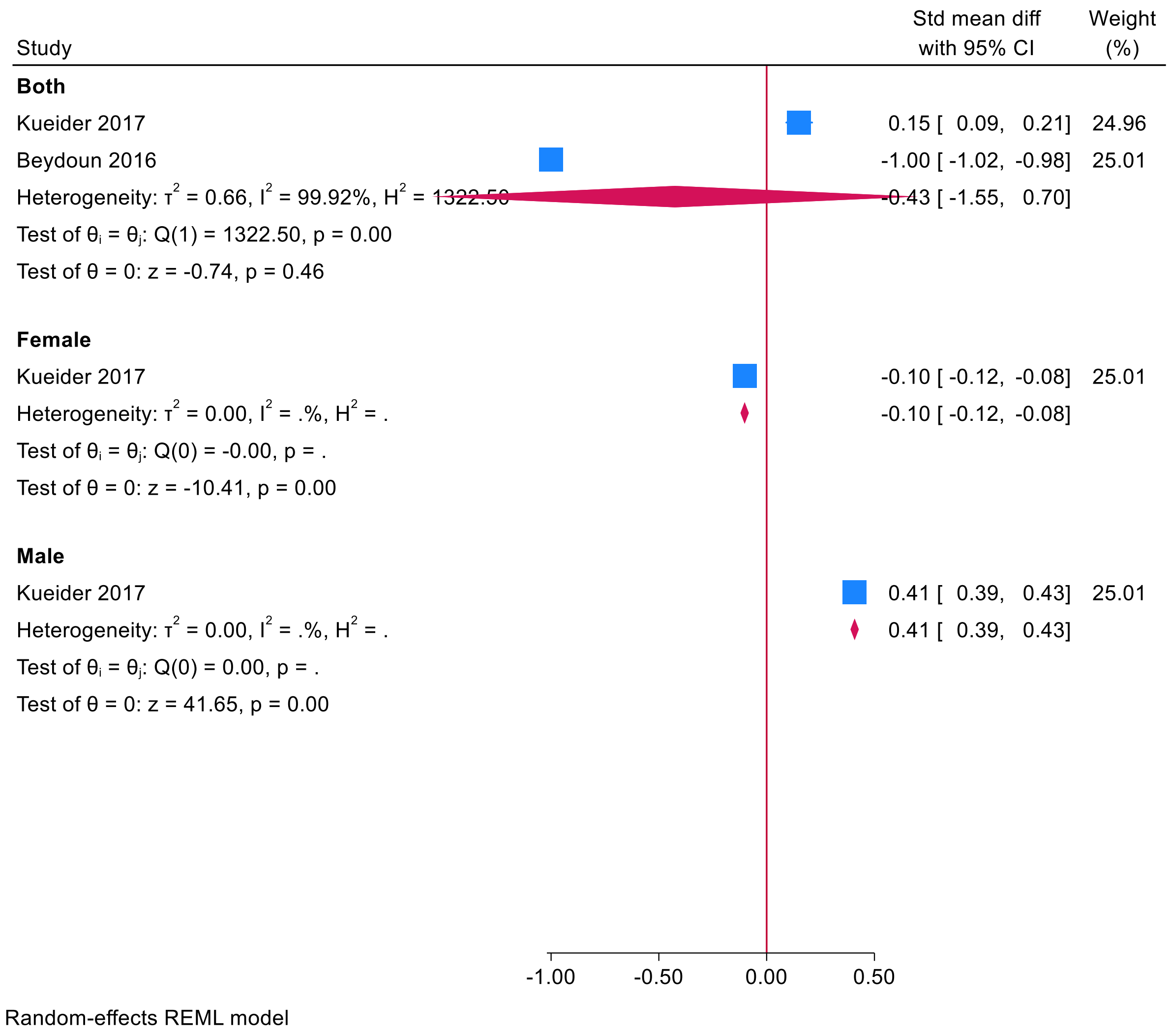

Figure S34: Forest plot for the association between continuous SUA levels and language for prospective cohort studies.

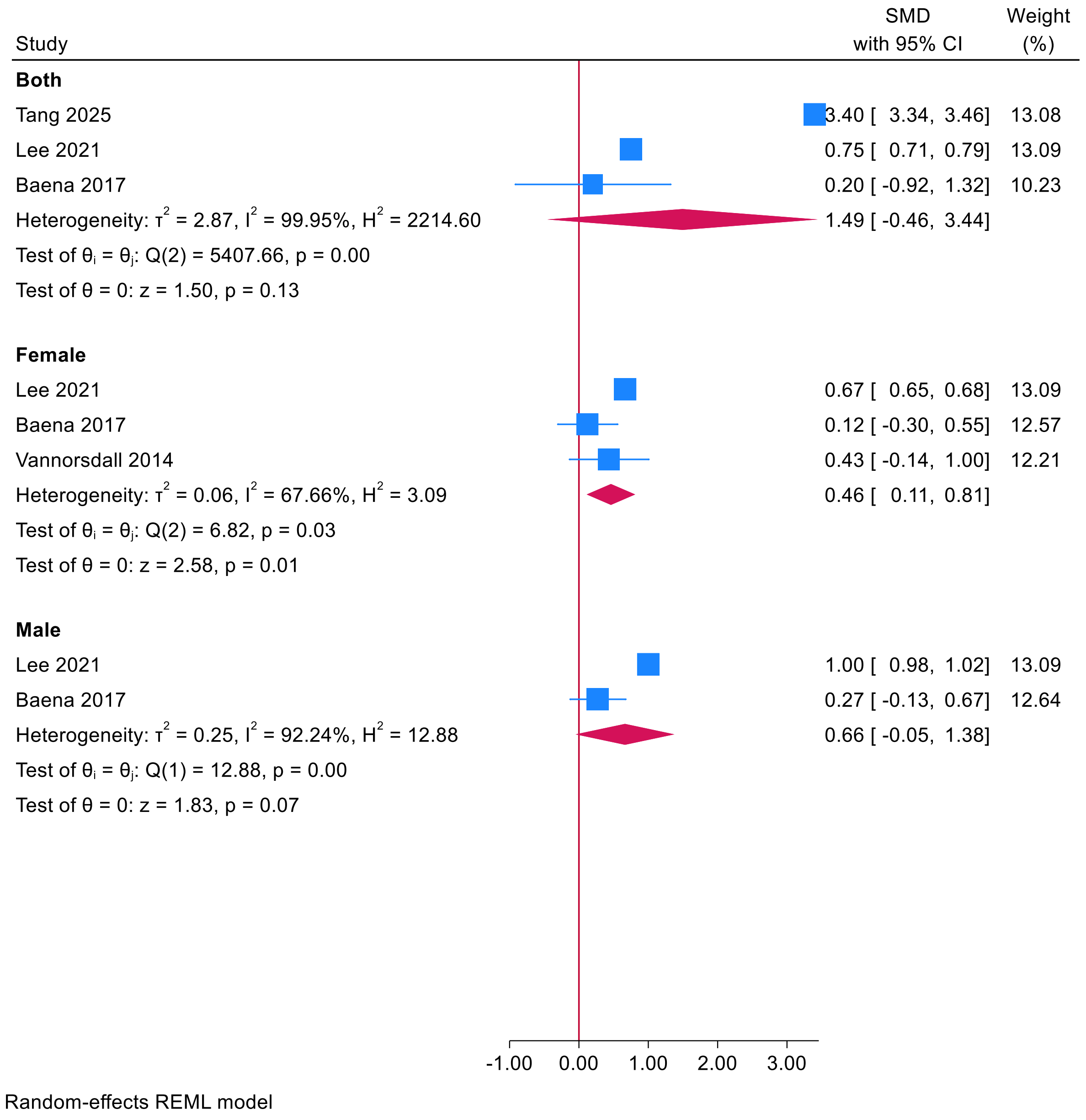

Figure S35A: Forest plot for the association between continuous SUA levels and language for cross-sectional studies.

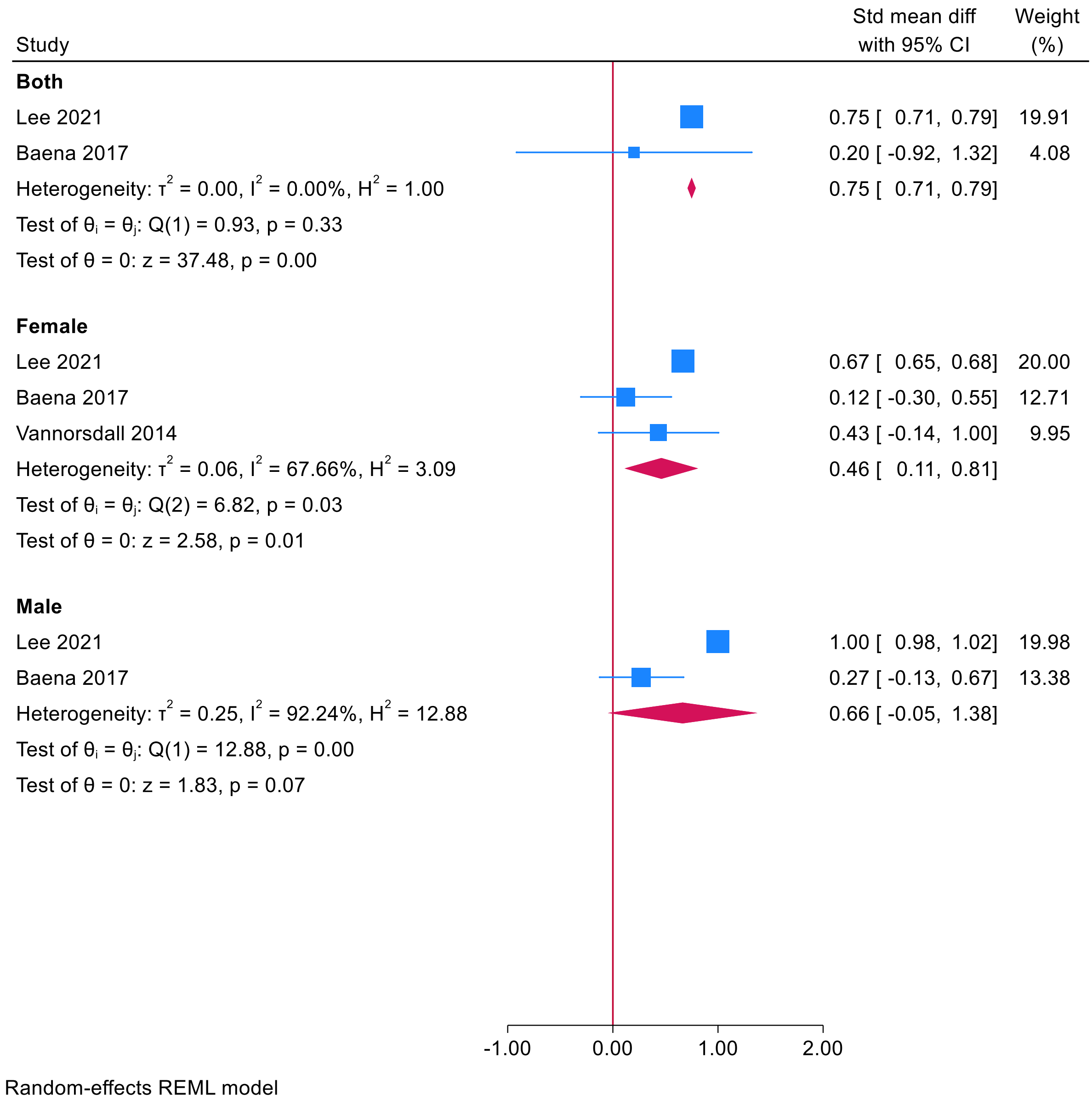

Figure S35B: Sensitivity analysis excluding a study (Tang et al. 2025; population on ALS) for cross-section study of continuous SUA level and language.

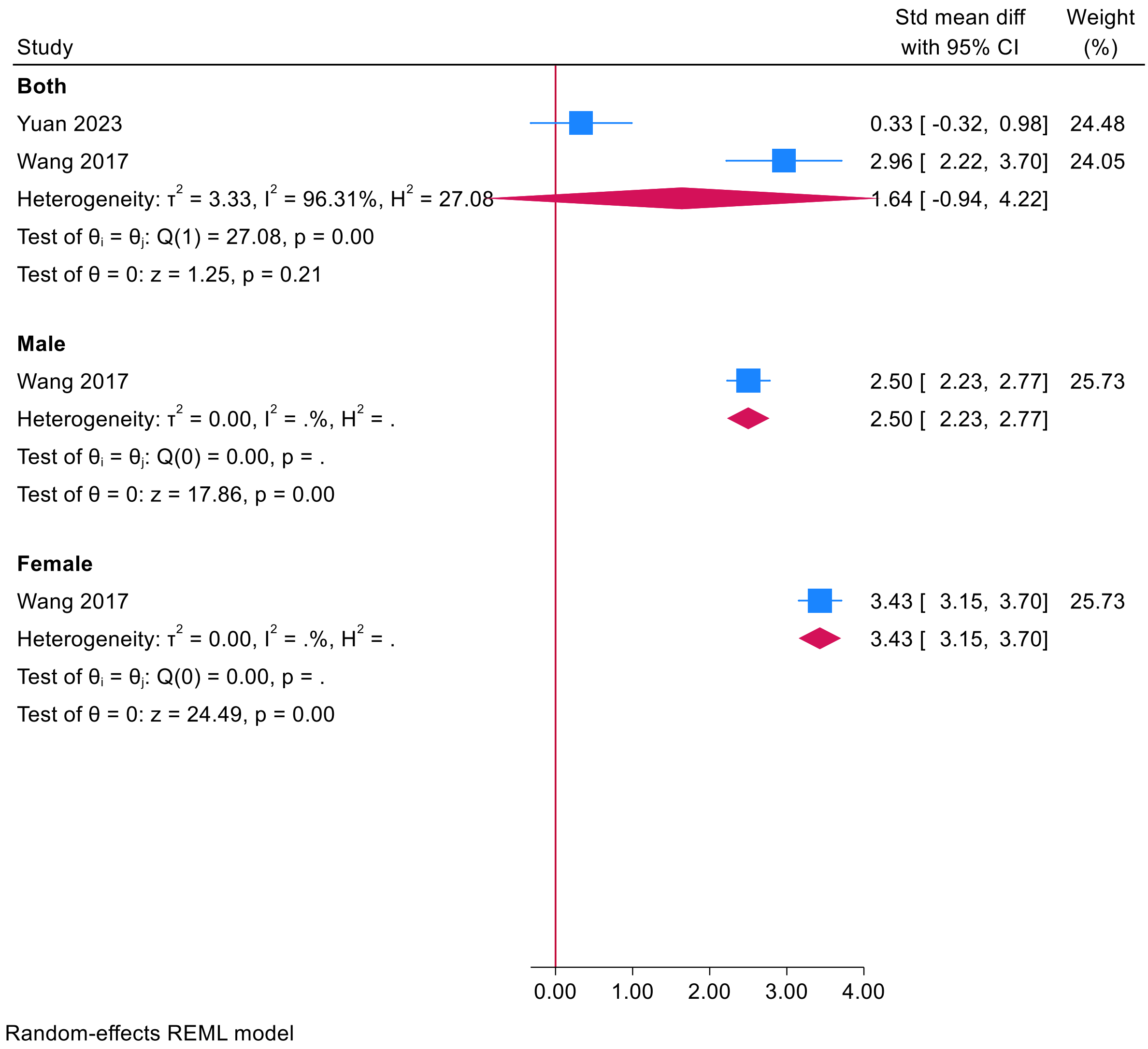

Figure S36: Forest plot for the association between categorical SUA levels (higher vs lower) and social cognition domain for cross-sectional studies.

References

1. Jiang L, Hu X, Jin J, et al. Inflammatory Risk Status Shapes the Association Between Uric Acid and Cognitive Function in Non-Hyperuricemia Middle Aged and Elderly. J Alzheimers Dis. 2024;97(1):249-257.
2. Zhang J, Jia X, Li Y, et al. The Association Between Serum Uric Acid and Cognitive Function Among Middle-Aged and Older Adults Without Hyperuricemia: The Mediating Role of Depressive Symptoms. J Gerontol A Biol Sci Med Sci. 2024;79(1).
3. Huang Y, Zhang S, Shen J, et al. Association of plasma uric acid levels with cognitive function among non-hyperuricemia adults: A prospective study. Clinical Nutrition. 2022;41(3):645-652.
4. Wang J, Jin R, Wu Z, et al. Moderate increase of serum uric acid within a normal range is associated with improved cognitive function in a non-normotensive population: A nationally representative cohort study. Front Aging Neurosci. 2022;14:944341.
5. Chen C, Li X, Lv Y, et al. High Blood Uric Acid Is Associated With Reduced Risks of Mild Cognitive Impairment Among Older Adults in China: A 9-Year Prospective Cohort Study. Frontiers in Aging Neuroscience. 2021;13:1-8.
6. Alam AB, Wu A, Power MC, et al. Associations of serum uric acid with incident dementia and cognitive decline in the ARIC-NCS cohort. J Neurol Sci. 2020;414:116866.
7. Kueider AM, An Y, Tanaka T, et al. Sex-Dependent Associations of Serum Uric Acid with Brain Function During Aging. J Alzheimers Dis. 2017;60(2):699-706.
8. Beydoun MA, Canas JA, Dore GA, et al. Serum Uric Acid and Its Association with Longitudinal Cognitive Change Among Urban Adults. J Alzheimers Dis. 2016;52(4):1415-1430.
9. Ye BS, Lee WW, Ham JH, et al. Does serum uric acid act as a modulator of cerebrospinal fluid Alzheimer's disease biomarker related cognitive decline? Eur J Neurol. 2016;23(5):948-957.
10. Euser SM, Hofman A, Westendorp RG, et al. Serum uric acid and cognitive function and dementia. Brain. 2009;132(Pt 2):377-382.
11. Tang J, Zhao Y, Chen Y, et al. White matter integrity mediated the effect of plasma uric acid levels on cognitive function in ALS patients. Brain Imaging Behav. 2025;19(3):678-689.
12. Khaled Y, Abdelhamid AA, Al-Mazroey H, et al. Higher serum uric acid is associated with poorer cognitive performance in healthy middle-aged people: a cross-sectional study. Intern Emerg Med. 2023;18(6):1701-1709.
13. Yuan Z, Liu H, Zhou R, et al. Association of serum uric acid and fasting plasma glucose with cognitive function: a cross-sectional study. BMC Geriatr. 2023;23(1):271.
14. Geng R, Zhang Y, Liu M, et al. Elevated serum uric acid is associated with cognitive improvement in older American adults: A large, population-based-analysis of the NHANES database. Front Aging Neurosci. 2022;14:1024415.
15. Lee YG, Park M, Jeong SH, et al. Effects of baseline serum uric acid and apolipoprotein E4 on longitudinal cognition and cerebral metabolism. Neurobiol Aging. 2021;106:223-231.
16. Sun J, Lv X, Gao X, et al. The association between serum uric acid level and the risk of cognitive impairment after ischemic stroke. Neurosci Lett. 2020;734:135098.
17. Baena CP, Suemoto CK, Barreto SM, et al. Serum uric acid is associated with better executive function in men but not in women: Baseline assessment of the ELSA-Brasil study. Exp Gerontol. 2017;92:82-86.
18. Wang T, Wu Y, Sun Y, et al. A Prospective Study on the Association between Uric Acid and Cognitive Function among Middle-Aged and Older Chinese. J Alzheimers Dis. 2017;58(1):79-86.
19. Liu M, Wang J, Zeng J, et al. Relationship between serum uric acid level and mild cognitive impairment in Chinese community elderly. BMC Neurol. 2017;17(1):146.
20. Perna L, Mons U, Schöttker B, et al. Association of cognitive function and serum uric acid: Are cardiovascular diseases a mediator among women? Exp Gerontol. 2016;81:37-41.
21. Molshatzki N, Weinstein G, Streifler JY, et al. Serum uric acid and subsequent cognitive performance in patients with pre-existing cardiovascular disease. PLoS One. 2015;10(3):e0120862.

22. Al-khateeb E, Althaher A, Al-khateeb M, et al. Relation between uric acid and Alzheimer's disease in elderly Jordanians. J Alzheimers Dis. 2015;44(3):859-865.
23. Vannorsdall TD, Kueider AM, Carlson MC, et al. Higher baseline serum uric acid is associated with poorer cognition but not rates of cognitive decline in women. Exp Gerontol. 2014;60:136-139.
24. Verhaaren BF, Vernooij MW, Dehghan A, et al. The relation of uric acid to brain atrophy and cognition: the Rotterdam Scan Study. Neuroepidemiology. 2013;41(1):29-34.
25. Wu Y, Zhang D, Pang Z, et al. Association of serum uric acid level with muscle strength and cognitive function among Chinese aged 50-74 years. Geriatr Gerontol Int. 2013;13(3):672-677.
26. Afsar B, Elsurer R, Covic A, et al. Relationship between uric acid and subtle cognitive dysfunction in chronic kidney disease. Am J Nephrol. 2011;34(1):49-54.
